# A four eigen-phase model of multi-omics unveils new insights into yeast metabolic cycle

**DOI:** 10.1093/nargab/lqaf022

**Published:** 2025-03-19

**Authors:** Linting Wang, Xiaojie Li, Jianhui Shi, Lei M Li

**Affiliations:** State Key Laboratory of Mathematical Science, Academy of Mathematics and Systems Science, Chinese Academy of Sciences, Beijing, 100190, China; School of Mathematical Sciences, University of the Chinese Academy of Sciences, Beijing, 101408, China; State Key Laboratory of Mathematical Science, Academy of Mathematics and Systems Science, Chinese Academy of Sciences, Beijing, 100190, China; School of Mathematical Sciences, University of the Chinese Academy of Sciences, Beijing, 101408, China; State Key Laboratory of Mathematical Science, Academy of Mathematics and Systems Science, Chinese Academy of Sciences, Beijing, 100190, China; School of Mathematical Sciences, University of the Chinese Academy of Sciences, Beijing, 101408, China; State Key Laboratory of Mathematical Science, Academy of Mathematics and Systems Science, Chinese Academy of Sciences, Beijing, 100190, China; School of Mathematical Sciences, University of the Chinese Academy of Sciences, Beijing, 101408, China

## Abstract

The yeast metabolic cycle (YMC), characterized by cyclic oscillations in transcripts and metabolites, is an ideal model for studying biological rhythms. Although multiple omics datasets on the YMC are available, a unified landscape for this process is missing. To address this gap, we integrated multi-omics datasets by singular value decompositions (SVDs), which stratify each dataset into two levels and define four eigen-phases: primary 1A/1B and secondary 2A/2B. The eigen-phases occur cyclically in the order 1B, 2A, 1A, and 2B, demonstrating an interplay of induction and repression: one eigen-phase induces the next one at a different level, while represses the other one at the same level. Distinct molecular characteristics were identified for each eigen-phase. Novel ones include the production and consumption of glycerol in eigen-phases 2A/2B, and the opposite regulation of ribosome biogenesis and aerobic respiration between 2A/2B. Moreover, we estimated the timing of multi-omics: histone modifications H3K9ac/H3K18ac precede mRNA transcription in ∼3 min, followed by metabolomic changes in ∼13 min. The transition to the next eigen-phase occurs roughly 38 min later. From epigenome H3K9ac/H3K18ac to metabolome, the eigen-entropy increases. This work provides a computational framework applicable to multi-omics data integration.

## Introduction

Biological rhythms, characterized by periodic patterns in behavior and physiology, are fundamental to diverse organisms ranging from yeast to humans. Human biological rhythms regulate essential functions such as sleep-wake cycles, hormonal levels, and metabolic activities. Disruption in these rhythms is linked to metabolic dysfunction, potentially resulting in diseases such as obesity, diabetes, and cancer. Thus, understanding human biological rhythms is an important area of current research. However, this area faces challenges due to the complexity of biological systems and the intricate interactions between environmental factors and genetic mechanisms.

The yeast metabolic cycle (YMC) of unicellular *Saccharomyces cerevisiae* serves as a minimal model for studying biological rhythms [1, 2]. Due to the conserved metabolic mechanisms between yeast and humans, studying the YMC can shed light on human biorhythms. Observed under nutrient-limited conditions, the YMC is characterized by periodic oscillations of dissolved oxygen levels, gene expression patterns, and metabolite concentrations. During the YMC, certain processes are confined to specific time windows, allowing cells to adapt to environmental factors. Based on gene expression clustering, the YMC is traditionally divided into three phases: oxidative (OX), reductive building (RB), and reductive charging (RC) [3].

Advances in biotechnology have generated multiple omics datasets of the YMC, including transcriptome, epigenome, and metabolome [3–12]. These datasets provided valuable molecular information regarding the YMC. For example, transcriptome and epigenome analyses showed that dynamic histone modification patterns correlate with gene expression [7], and identified certain modifications and transcription factors (TFs) [9]. Metabolomic studies revealed periodic changes in intracellular metabolite concentrations, highlighting pivotal metabolites in the YMC [5, 6, 11]. However, most studies focused on single omics data while only a few integrated two datasets [7, 9, 13, 14]. Thus, a computational framework is highly desirable to synthesize a unified landscape of the YMC by integrating all available omics data.

Gene expression variance, which quantifies the variability in the expression levels of a single gene across different cells or individuals, offers critical insights into processes such as cell development, disease, and adaptation [15]. Genes with low variance are often associated with fundamental cellular processes such as housekeeping functions [15], while those with high variance are commonly involved in immune responses, environmental interactions, and stress responses [16]. Despite their importance, the gene expression variances have not been fully considered in the YMC data analysis. Moreover, each omics dataset could have different molecular signal variances, whose quantification remains another interesting problem to be answered.

Other than molecular signal variances, the timing of various omics events is a key to understanding the dynamics of the YMC. Previous studies have primarily focused on the relative timing of epigenetic modifications and transcripts for several gene clusters [7]. While informative, these studies do not fully address the overall timing across multi-omics of the YMC. Filling this gap could enhance our understanding of the temporal organization of omics in the YMC.

**Figure 1.**
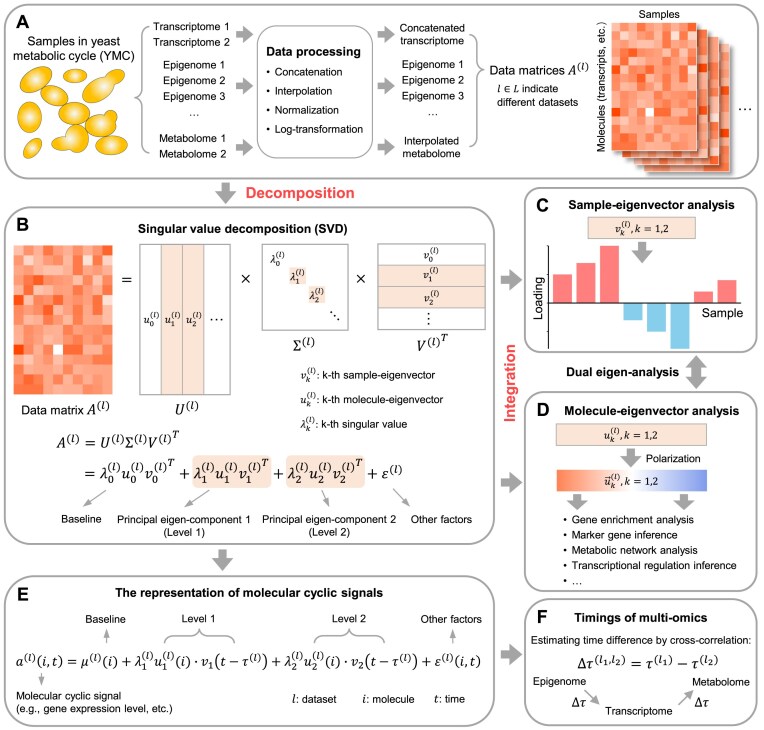
The workflow of the multi-omics data synthesis analysis. (**A**) Raw omics datasets are preprocessed using various methods to generate multiple data matrices. (**B**) Each data matrix is stratified by SVD into multiple principal eigen-components, referred to as levels. Each level comprises a singular value λ_k_, and a pair of sample-eigenvector *v_k_* and molecule-eigenvector *u_k_*. The top two levels (marked with a shaded background) contribute mainly to the data. The 0-th level represents the baseline, while the rest levels are categorized as other factors. (**C**) The positive and negative loadings of sample-eigenvectors show temporal patterns during the YMC. (**D**) Molecule-eigenvectors are polarized by sorting their loadings. Various analyses are then applied to uncover molecular signatures associated with the positive and negative poles of each molecule-eigenvector. The dual correspondence between sample- and molecule-eigenvector is illustrated in Supplementary Fig. S1. (**E**) SVD offers a representation of molecular cyclic signals. This includes a baseline, two levels, and other factors. Each level consists of a singular value λ, a molecule loading function *u(i)*, and a periodic function *v(t)*. (**F**) Time differences between omics data, represented by the shift parameter in the periodic functions, are estimated using cross-correlation.

To address these gaps, we developed a novel synthesis approach to integrating YMC multi-omics datasets (Fig. 1). Motivated by our previous work [17, 18], the approach is based on singular value decomposition (SVD) and dual eigen-analysis. It involves several steps. First, each omics dataset was arranged in a matrix followed by preprocessing methods such as normalization (Fig. 1A). Second, these matrices were stratified by SVD into two principal eigen-components, referred to by their levels, each comprising a singular value and a pair of sample- and molecule-eigenvectors (Fig. 1B). Third, while the sample- and molecule-eigenvectors respectively revealed sample eigen-phases and molecular characteristics (Fig. 1C and D), their associations were identified by dual eigen-analysis (Supplementary Fig. S1).

The proposed synthesis approach uncovered several novel findings. First, a four-eigen-phase model was identified in these datasets. The eigen-phases—referred to as primary 1A/1B and secondary 2A/2B—demonstrate an interplay of induction and repression. One eigen-phase induces the next one chronologically, while two eigen-phases at the same level reciprocal repress each other. Each eigen-phase was characterized by molecular features, including signature pathways, marker genes, key metabolites, and TFs. Specifically, eigen-phase 1A was characterized by translation and amino acid metabolism, 1B by degradation mechanisms and stress responses, 2A by ribosome biogenesis, and 2B by aerobic respiration and mitochondrial activities. Second, our model also provided a mathematical representation of molecular cyclic signals during the YMC (Fig. 1E). This representation decomposes the molecular signal variances into two levels, reflecting the intrinsic hierarchical structure of the YMC. Third, the average timing was estimated across multi-omics in the temporal order of the epigenome for H3K9ac and H3K18ac, transcriptome, and metabolome. The timing reflects an adaptive regulatory mechanism to utilize the nutrition available from the environment for survival.

## Materials and methods

### Transcriptome, epigenome, and metabolome data of YMC

The basic information on the omics datasets used in this study is presented in Supplementary Table S1. All datasets were obtained from public databases or directly from the articles.

The transcriptomic microarray dataset of gene expression was obtained from the GEO repository under accession number GSE3431 [3]. In the referenced study, messenger RNA (mRNA) from YMC yeast samples was collected at 36 time points over three consecutive cycles, with ∼25-min intervals between adjacent time points. The mRNA abundance was quantified using the Affymetrix Yeast Genome S98 Array.

The transcriptomic RNA-seq dataset of gene expression was obtained from the GEO repository under accession number GSE52339 [7]. In the referenced study, mRNA from YMC yeast samples was collected at 16 distinct time points during a single cycle. In our study, the RNA-seq raw reads underwent quality filtering using FastQC (https://www.bioinformatics.babraham.ac.uk/projects/fastqc/) and were then trimmed using TrimGalore (https://www.bioinformatics.babraham.ac.uk/projects/trim_galore/) to remove low quality reads. Next, sequencing reads were aligned to the *Saccharomyces cerevisiae* reference genome sacCer3 in the UCSC database (https://hgdownload.soe.ucsc.edu/goldenPath/sacCer3/bigZips/) using Hisat2 [19]. Finally, featureCounts [20] was used to quantify the expression levels of each gene in the RNA-seq data.

The epigenetic ChIP-seq dataset of histone modifications was obtained from the GEO repository under accession numbers GSE52339 and GSE118889 [7, 9]. In the referenced studies, histone modifications were identified using antibodies against eight marks: H3K4me3, H3K9ac, H3K14ac, H3K18ac, H3K36me3, H3K56ac, H4K5ac, and H4K16ac. The DNA bound to histones in YMC yeast samples was collected at 16 distinct time points during a single cycle. In our study, the ChIP-seq raw reads underwent quality filtering using FastQC and were then trimmed using TrimGalore to remove low-quality reads. Some samples of histone modifications H3K36me3 and H4K16ac were removed due to low sequencing quality. Next, sequencing reads were aligned to the *S. cerevisiae* reference genome sacCer3 in the UCSC database using Bowtie [21] due to the short read lengths typical of ChIP-seq data.

ChIP-seq signals of each histone modification sample were evaluated in the same way as the methods in the original studies [7, 9]. Signal strength was defined as the number of ChIP-seq reads mapped to the predefined genomic regions of each gene. These regions were selected based on the distribution of signals, c.f. Fig. S5A of [7]. Specifically, the region of −100 bp to 400 bp from the transcription start site (TSS) was used for H3K9ac, H3K14ac, H3K18ac, H3K56ac, H4K5ac, and H3K4me3, while the region from TSS to transcription end site (TES) was used for H3K36me3 and H4K16ac. The read counts were quantified using featureCounts with the -O parameter option.

The metabolome of intracellular metabolite concentrations was obtained from the supplementary information provided in [6]. In the referenced study, metabolites from YMC yeast samples were collected at 24 time points over two consecutive cycles. Liquid chromatography tandem mass spectrometry (LC-MS) and comprehensive 2D gas chromatography time-of-flight mass spectrometry (GC-TOFMS) were used to monitor the intracellular concentrations of ∼150 metabolites. In addition, the extracellular concentrations of ethanol and acetate were obtained from [3].

### Normalization of omics data

The transcriptomic microarray dataset was normalized using sub-sub normalization [22], while the RNA-seq sequencing reads were normalized using MUREN [23]. The reference samples for normalization were selected based on the largest sum of skewness in pairwise difference. Subsequently, logarithmic transformation was applied to the normalized data.

Similarly, the epigenetic ChIP-seq signal strength was normalized using MUREN in the same way as described above. The quality of normalization was demonstrated by near-zero modes of densities of pairwise difference [23] (Supplementary Fig. S2). The logarithmic transformation was applied.

The metabolome lacked a specific normalization method. However, the different scales of LC-MS and GC-TOFMS need to be considered. In our study, logarithmic transformation was used to mitigate the scale effects.

### SVD and dual eigen-analysis of omics data

We took the SVD and dual eigen-analysis as the main approach for this study. It was introduced in our previous studies [17, 18, 24] and has demonstrated effectiveness in processing high-dimensional data matrices, especially for normalized transcriptomic data [17]. A schematic representation of SVD and dual eigen-analysis is provided in Fig. 1B and Supplementary Fig. S1.

Each omics dataset was preprocessed into a data matrix. Here, we consider an example matrix denoted by $A = [ {{a_{ij}}} ]$, where $i\; = \;1,\;2,\;\ldots,\;m$ and$\;j\; = \;1,\;2,\;\ldots,\;n$. Here, $m$ represents the number of molecules (e.g. genes and metabolites), and $n$ represents the number of samples. The SVD of matrix $A$ is expressed as follows:


\begin{eqnarray*}A = \mathop \sum \limits_{k = 0}^{s - 1} {\lambda _k}{u_k}v_k^T,\end{eqnarray*}

where $\{ {{\lambda _k}} \}$ are positive and sequentially decreasing singular values; the molecule-eigenvectors ${u_k}$ is denoted by ${[ {{u_{k1}},\;{u_{k2}},\;\ldots,\;{u_{km}}} ]^T}$, and the sample-eigenvectors ${v_k}$ is denoted by ${[ {{v_{k1}},\;{v_{k2}},\;\ldots,\;{v_{kn}}} ]^T}$; $\{ {{u_k}} \}$ are mutually orthogonal and so are $\{ {{v_k}} \}$; and $s$ is the rank of $A$.

The SVD stratifies matrix $A$ into multiple principal eigen-components. The *k*-th principal eigen-component, referred to as level *k*, comprises ${\lambda _k}$, ${u_k}$, and ${v_k}$. The *k*-th molecule-eigenvector ${u_k}$ is composed of the weights of all molecules, referred to as molecule loadings. Similarly, the *k*-th sample-eigenvector ${v_k}$ is composed of the weights of all samples, referred to as sample loadings.

Notably, the level with the largest singular value typically represents the baseline of data, and its index is therefore set to zero. Specifically, after normalization, the loadings of the 0-th sample-eigenvector ${v_0}$ are nearly identical, as shown in Supplementary Fig. S3. Because ${v_0}$ is a unit vector, we have ${v_0} \cong \frac{1}{{\sqrt n }}{1_n}$, where ${1_n}\; = \;{[ {1,\;1,\;\ldots,\;1} ]^T}$ represents a vector with all elements equal to 1. We also have ${\lambda _0}{u_0} = A{v_0} \cong \frac{1}{{\sqrt n }}A{1_n} = \sqrt n \mu$, where $\mu = \frac{1}{n}A{1_n} = {[ {{\mu _1},\;{\mu _k},\;\ldots,\;{\mu _m}} ]^T}$, and ${\mu _i} = \;\frac{1}{n}\mathop \sum \limits_{j = 1}^n {a_{ij}}$ represents the average quantity of molecule $i$ across all samples. Then the 0-th eigen matrix is expressed as:


\begin{eqnarray*}{\lambda _0}{u_0}v_0^T \cong \mu 1_n^T.\end{eqnarray*}

Therefore, the 0-th principal eigen-component exclusively represents the average matrix, considered the baseline of the data. Then we define the matrix after removing the baseline as $\bar A = A - {\lambda _0}{u_0}v_0^T$. This matrix approximates what is usually referred to as the centralized matrix.

The singular value plays an important role in SVD. For $k > 0$, we have the equation:


\begin{eqnarray*}\lambda _k^2 = ||\bar A{v_k}|{|^2} = ||{\lambda _k}{u_k}v_k^T|{|^2}.\end{eqnarray*}

It indicates that the square of the singular value $\lambda _k^2$ quantifies the variance of the matrix $\bar A$ in the direction of the sample-eigenvector ${v_k}$. In addition, the sum of the square of singular values (except the one at level 0) equals the square of the Frobenius norm of matrix $\bar A$. That is,


\begin{eqnarray*}\sum\nolimits_{k = 1}^{s - 1} {\lambda _k^2 = ||\bar A||_F^2} ,\end{eqnarray*}

where $|| \cdot |{|_F}$ represents the Frobenius norm. Therefore, $||\bar A||_F^2$ quantifies the total variance of the matrix, and $\lambda _k^2$ quantifies the contribution of level *k* to the total variance. We use the ratio $\frac{{\lambda _k^2}}{{\mathop \sum \nolimits_{k = 1}^{s - 1} \lambda _k^2}}$ to quantify the relative contribution of each level.

Consistent with our previous studies [17], the loadings of sample-eigenvectors ${v_k}$ is an empirical contrast. Specifically, $\mathop \sum \limits_{j = 1}^n {v_{kj}} \cong 0$, for $k > 0$, which indicates the sum of loadings for positive and negative samples is nearly equal. According to the properties of SVD, it is established that ${\lambda _k}{u_k} = A{v_k}$. Therefore, the loading ${u_{ki}}$ of molecule $i$ is expressed as


\begin{eqnarray*}{u_{ki}} = \frac{{\mathop \sum \nolimits_{j = 1}^n {a_{ij}}{v_{kj}}}}{{{\lambda _k}}},\end{eqnarray*}

where numerator $\mathop \sum \limits_{j = 1}^n {a_{ij}}{v_{kj}}$ represent the weighted sum of values for molecule $i$ across all samples. Thus, within level *k*, a higher molecule loading ${u_{ki}}$ correlates with higher values ${a_{ij}}$ and higher sample loading ${v_{kj}}$. In addition, molecule loading ${u_{ki}}$ can be regarded as the differential profiles of the molecule under the corresponding sample weights.

The above investigations culminated in our dual eigen-analysis [17, 18] (Supplementary Fig. S1). Specifically, in the first step, ${u_k}$ and ${v_k}$ are polarized into ${\vec u_k}$ and ${\vec v_k}$ by arranging their loadings in ascending order. Next, we identify the associations between molecules at both poles of ${\vec u_k}$ together with their molecular attributes, and samples at both poles of ${v_k}$ together with their timing attributes. Finally, molecules at the positive pole of ${\vec u_k}$ exhibit up-regulation in samples with positive loadings and down-regulation in samples with negative loadings. Conversely, molecules at the negative pole of ${\vec u_k}$ exhibit down-regulation in samples with positive loadings, and up-regulation in samples with negative loadings.

### The decomposition formula of molecular cyclic signals

From the SVD of matrix $A = [ {{a_{ij}}} ]$, each element can be decomposed as follows:


\begin{eqnarray*}{a_{ij}} = \mathop \sum \limits_{k = 0}^{s - 1} {\lambda _k}{u_{ki}}{v_{kj}}.\end{eqnarray*}

Given that many molecules are periodic during the YMC, we assume that the cyclic signal of molecule $i$ follows a periodic function $a( {i,t} )$, where $t$ represents time. Assuming sample $j$ was collected at the time ${t_j}$, the following equation can be established:


\begin{eqnarray*}a\left( {i,{t_j}} \right) = {a_{ij}} = \mathop \sum \limits_{k = 0}^{s - 1} {\lambda _k}{u_{ki}}{v_{kj}} = \mathop \sum \limits_{k = 0}^{s - 1} {\lambda _k}{u_k}\left( i \right)\cdot{v_k}\left( {{t_j}} \right),\end{eqnarray*}

where ${u_k}( \cdot )$ is the function of molecule loading, ${v_k}( t )$ is a continuous periodic function that satisfies ${v_k}( {{t_j}} ) = {v_{kj}}$, for any $j$.

As we demonstrated in the previous subsection, the 0-th principal eigen-component represents the average matrix, and we established that ${\lambda _0}{u_{0i}}{v_{0j}} \cong {\mu _i}$, where ${\mu _i} = \frac{1}{n}\mathop \sum \limits_{j = 1}^n {a_{ij}}$, represents the average quantity of molecule $i$ across all samples. We now denote ${\mu _i}$ as a function $\mu ( i )$. Moreover, all other minor eigen-components beyond level 2 are aggregated into a function $\varepsilon ( {i,t} )$. Finally, we come to the decomposition formula:


\begin{eqnarray*}a\left( {i,t} \right) = \mu \left( i \right) + {\lambda _1}{u_1}\left( i \right)\cdot{v_1}\left( t \right) + {\lambda _2}{u_2}\left( i \right)\cdot{v_2}\left( t \right) + \varepsilon \left( {i,t} \right).\end{eqnarray*}

In this formula, the singular values ${\lambda _1}\;$and ${\lambda _2}$ quantify the molecular signal variances of levels 1 and 2, respectively, whereas ${v_1}( t )$ and ${v_2}( t )$ are periodic functions.

To distinguish different omic data, we further label the decomposition for each data by $l \in L$:


\begin{eqnarray*}{a^{\left( l \right)}}\left( {i,t} \right) &=& {\mu ^{\left( l \right)}}\left( i \right) + \lambda _1^{\left( l \right)}u_1^{\left( l \right)}\left( i \right) \cdot {v_1}\left( {t - {\tau ^{\left( l \right)}}} \right) \nonumber\\ &+& \lambda _2^{\left( l \right)}u_2^{\left( l \right)}\left( i \right) \cdot {v_2}\left( {t - {\tau ^{\left( l \right)}}} \right) + {\varepsilon ^{\left( l \right)}}\left( {i,t} \right)\end{eqnarray*}

where ${\tau ^{( l )}}$ represents the time lag relative to a reference, which the transcriptome is used in this report, c.f. Fig. 1E.

### Concatenation of transcriptome data matrices

To obtain more stable and comprehensive results, we integrated the two transcriptomic data matrices from [3, 7] by concatenation, a method proposed in our previous study [17]. The concatenation scheme is presented in Supplementary Fig. S4.

The two transcriptomic data matrices with common genes are denoted as ${A_1}$ and ${A_2}$. Before concatenation, several preprocessing steps were undertaken. First, their scales were aligned by removing baselines, specifically by subtracting the 0-th eigen matrix determined by SVD. Second, their variations were equalized by adjusting the Frobenius norm, ensuring the densities of the two matrices approximated each other (Supplementary Fig. S5). Third, to mitigate the impact of varying sample sizes, each matrix was divided by the square root of its sample number.

As a result, we obtained the preprocessed transcriptomic data matrices $\widetilde {{A_1}}$ and $\widetilde {{A_2}}$. Next, these matrices were concatenated to form a single matrix


\begin{eqnarray*}\tilde A = \left[ {\begin{array}{*{20}{c}} {\begin{array}{*{20}{c}} {\widetilde {{A_1}}\;\widetilde {\;{A_2}}} \end{array}} \end{array}} \right].\end{eqnarray*}

Finally, SVD and dual eigen-analysis were performed:


\begin{eqnarray*}\tilde A = \mathop \sum \limits_{k = 1}^{\tilde s} {\tilde \sigma _k}{\tilde u_k}\tilde v_k^T\end{eqnarray*}

where $\tilde s$ is the rank of $\tilde A$, and the sample-eigenvector ${\tilde v_k} = \left[ {\begin{array}{@{}*{1}{c}@{}} {\tilde {{v_1}}_k}\\ {\tilde {{v_2}}_k} \end{array}} \right]$ can be partitioned into components corresponding to samples from each dataset. It is important to note that the indexing begins at one, not zero, reflecting the prior removal of the baseline.

### Alignment of omics data by dissolved oxygen concentration curves

Oxygen concentrations were extracted from the curves in the original studies [3, 6, 7, 9]. Specifically, a Python script was used to extract the pixel coordinates from oxygen concentration curves. The *X*- and *Y*-axis coordinates were extracted to calibrate the time and oxygen concentration values of each pixel. When multiple pixels shared the same *X*-axis coordinates due to curve width, their *Y*-axis coordinates were averaged. The sampling time points for the omics datasets were directly obtained from the original studies [3, 6, 7, 9]. The extracted oxygen concentration curves, along with sampling time points for each dataset, are shown in Supplementary Fig. S6.

The derivative dynamic time warping (DDTW) method [25] was selected to align the oxygen concentration curves from different datasets. This method is based on dynamic time warping (DTW), which is widely used for the alignment of signal curves. Compared with DTW, DDTW captures the changing trend of the curves more effectively, achieving a more uniform alignment of the oxygen concentration curves.

Using DDTW, the metabolomic dataset was aligned to the transcriptomic microarray dataset because both span multiple YMC cycles (Supplementary Fig. S7). On the other hand, the epigenetic dataset was aligned with the transcriptomic RNA-seq dataset, as both were sampled within a single cycle. After alignment, the samples from the various omics datasets could be represented on a unified timeline.

Following alignment, metabolome data derived from various methods were integrated into a single data matrix. Specifically, metabolite data from GC-TOFMS was mapped to the time points of LC-MS samples using linear interpolation. Consequently, data from both GC-TOFMS and LC-MS shared the same sampling time points, resulting in an integrated data matrix.

### Cross-correlation between sample eigenvectors

The maximum cross-correlation was used to quantify the similarity between two time series. In this study, it was used to evaluate the consistency between sample-eigenvectors across datasets. To achieve this, a three-step process was employed. First, a natural spline interpolation was applied to resampling the loading curves at a high resolution of 0.001 h, ensuring that these curves share the same time points. Second, one loading curve was shifted by a time lag in relation to the other, and the cross-correlation was computed at each shift. Finally, the maximum cross-correlation was taken to be the similarity between two time series.

### Estimation of time differences across various omics data

By comparing the loading curves of sample-eigenvectors from different omics datasets after alignment by oxygen concentrations, we observed time differences between these datasets. Mathematically, the time difference between two datasets ${l_1},\;{l_2}$ can be expressed in the following formula:


\begin{eqnarray*}\Delta {\tau ^{({l_1},{l_2})}} = {\tau ^{({l_1})}} - {\tau ^{\left( {{l_2}} \right)}},\end{eqnarray*}

where ${\tau ^{({l_1})}}$ and ${\tau ^{( {{l_2}} )}}$ are the time lags in the decomposition formula for each dataset, c.f. Fig. 1F.

The estimate of time difference between two series is taken to be the time lag corresponding to the maximum cross-correlation between two sample eigenvectors. The calculation steps for this process were described in detail in the previous Method subsection.

To evaluate the robustness of our time difference estimation method, we introduced normally distributed random perturbations to simulate measurement noise and data uncertainty. The standard deviation of the perturbations was set to 10% of the original data’s standard deviation.

### Gene set enrichment by the Wilcoxon scoring method

Enrichment analysis of the polarized gene-eigenvector ${\vec u_k}$ was performed using the Wilcoxon scoring method [26]. This method was originally proposed for analyzing gene expression differential profiles. In this study, gene loadings, as discussed above, were regarded as the weighted gene expression differential profiles and were used in the analysis. Gene sets were from Gene Ontology (GO) annotations [27]. Only gene sets containing five or more genes were included in the analysis.

### Identification of marker gene by randomization

The significance of each gene in gene-eigenvector ${u_k}$ was assessed by the randomization of dual sample-eigenvector ${v_k}$, using the property of ${\lambda _k}{u_k} = A{v_k}$. And the significant genes were referred to as marker genes. Specifically, as the *k*-th sample-eigenvector ${v_k}$ satisfies


\begin{eqnarray*}{v_k} = \mathop {argmax}\limits_{{{\left| {\left| x \right|} \right|}_2} = 1,\;x\cdot{v_l} = 0,l \le k} {\left| {\left| {Ax} \right|} \right|_2},\end{eqnarray*}

we generate a random sample-eigenvector ${v_k}^r$ satisfies


\begin{eqnarray*}{\left| {\left| {{v_k}^r} \right|} \right|_2} = 1,\;{v_k}^r\cdot{v_l} = 0,l \le k.\end{eqnarray*}

The dual random gene-eigenvector ${u_k}^r$ is then obtained as


\begin{eqnarray*}{u_k}^r = \frac{{A\cdot{v_k}^r}}{{{{\left| {\left| {A\cdot{v_k}^r} \right|} \right|}_2}}}.\end{eqnarray*}

Assuming we repeat this randomization for *R* times. Denote ${u_k}^r = {[ {{u_{k1}}^r,\;{u_{k2}}^r,\;\ldots,\;{u_{km}}^r} ]^T},\;r = 1,\ldots,R$. Then for gene $i$ in the *k*-th gene-eigenvector, we calculate its *P*-value by comparing its observed loading ${u_{ki}}$ to its distribution under the null hypothesis, represented by ${\{ {{u_{ki}}^r} \}_r}$. That is,


\begin{eqnarray*}{p_{ki}} = \left\{ {\begin{array}{@{}*{1}{c}@{}} {\frac{{\# {{\left\{ {{u_{ki}}^r \ge {u_{ki}}} \right\}}_r}}}{R},\;{u_{ki}} > 0}\\ {\frac{{\# {{\left\{ {{u_{ki}}^r \le {u_{ki}}} \right\}}_r}}}{R},\;{u_{ki}} < 0} \end{array}} \right.,\end{eqnarray*}

where $\# {\{ {{u_{ki}}^r \ge {u_{ki}}} \}_r}$ represents the count of *r* that satisfies ${u_{ki}}^r \ge {u_{ki}}$.

Finally, we identify marker genes in each gene-eigenvector by establishing thresholds for *P*-values and loadings.

### Statistical inference of *cis–trans* transcriptional regulation by BASE2.0

The method BASE2.0 [28, 29] was employed to statistically infer *cis–trans* transcriptional regulations underlying the gene-eigenvector ${u_k}$. When applying BASE2.0, the binding strength between the factor and the regulatory region of a gene needed to be defined.

To infer the regulation of TFs, the binding strength was defined using the motif frequency of TFs in the regulatory region of genes [28, 29]. In this study, the motif frequencies were calculated using the following steps. First, the position weight matrices of 186 *cis-*regulatory motifs, corresponding to 172 TFs of *S. cerevisiae*, were obtained from the Fungi dataset within the JASPAR database [30]. Second, the regulatory region was defined as the 500 bp upstream from the TSS of each gene. The sequences of these regions were sourced from the yeast whole genome via the Saccharomyces Genome Database (SGD) [31]. The region length was carefully selected based on the distribution of TF-binding sites reported in [32]. Third, the motif frequency within the gene regulatory region was calculated using FIMO in the MEME suite [33, 34].

## Results

### Decomposition of YMC multi-omics datasets through SVD

The YMC omics datasets used in this study are summarized in Supplementary Table S1. These datasets cover one or more cycles with multiple sampling time points. Each dataset was initially processed into matrices (Fig. 1A). Specifically, the transcriptome matrix quantified gene expression profiles; the epigenome matrix of each histone modification quantified its signal strength, and the metabolome matrix quantified metabolite concentrations. To make the results more robust, we concatenated the transcriptome matrices and aligned the metabolome ones. The effectiveness of these integration methods was evaluated in the Supplementary Text and Supplementary Fig. S8.

Next, each data matrix was stratified using SVD into multiple principal eigen-components (Fig. 1B). Each principal eigen-component comprised a singular value and a pair of corresponding sample- and molecule-eigenvectors. The elements of these eigenvectors are referred to as sample or molecule loadings, which represent their weights in this eigen-component. These eigen-components, referred to as levels, were arranged in descending order based on their singular values. Notably, the level with the largest singular value represents the data baseline, as its sample loadings are nearly equal (“Materials and methods” section and Supplementary Fig. S3). Therefore, we indexed it as zero and excluded it from subsequent analysis.

### Four eigen-phases of the YMC: primary 1A/1B and secondary 2A/2B

The relative contribution of each level to the data was quantified by the ratios of squared singular values (“Materials and methods” section). The results of each omics dataset can be found in Supplementary Fig. S9. In the transcriptome, epigenome (for H3K9ac, H3K18ac, H3K56ac, and H4K5ac), and metabolome, the top two levels (1 and 2) collectively contributed over 50% of the total variance.

**Figure 2.**
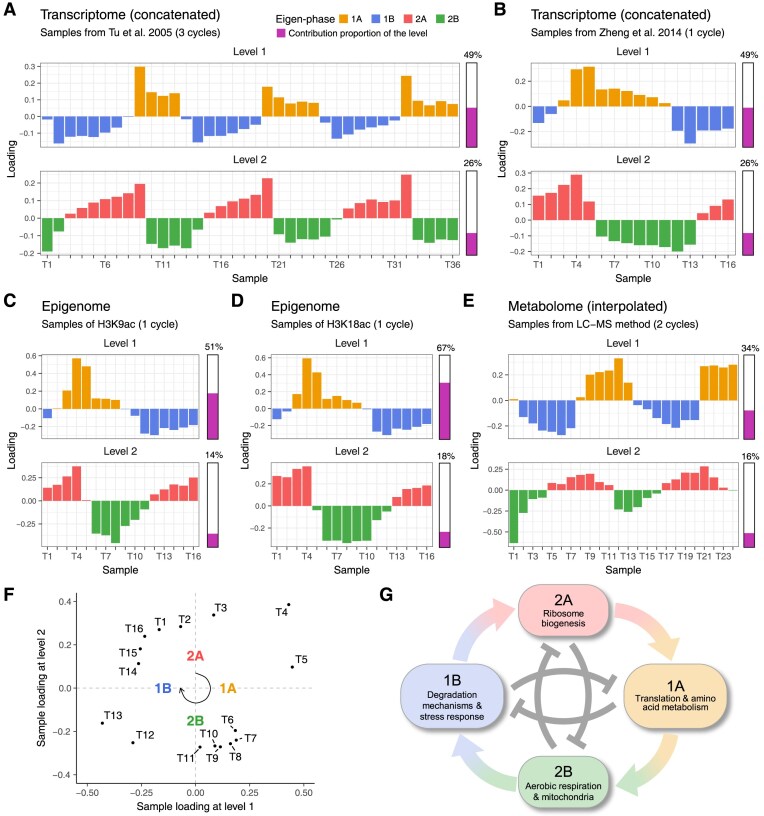
(**A–** **E**) Sample loadings at the top two levels define four YMC eigen-phases for different omics datasets. The samples are arranged chronologically. These four eigen-phases are termed 1A, 1B, 2A, and 2B, corresponding to positive and negative sample loadings at levels 1 and 2. The bar on the right of each subplot shows its relative contribution, calculated as the ratio of its squared singular value to the total sum. (**F**) Scatter plot of sample loadings from transcriptomic RNA-seq data at the top two levels. The temporal order of the samples shows that the four eigen-phases cycle in the sequence of 1B, 2A, 1A, and 2B. (**G**) The schematic diagram of the induction and repression between eigen-phases. Each node represents an eigen-phase with its signature biological processes. The arrows represent the inductive relationship from one eigen-phase to the next. The inhibitory curves in the central represent reciprocal repressions between the eigen-phases at the same level.

At the top two levels of sample-eigenvectors from the aforementioned omics datasets, four YMC eigen-phases were identified: 1A, 1B, 2A, and 2B (Fig. 2A–E and Supplementary Fig. S10). One such eigen-phase is defined by samples with the same loading signs, representing a time period with shared characteristics in the YMC. Across multi-omics, these eigen-phases exhibited a similar pattern, cycling in the sequence of 1B, 2A, 1A, and 2B (Fig. 2F). Compared with the traditional three-phase division [3], our result offers several novel insights.

The first insight is that these eigen-phases are defined at two levels, stratifying their roles in YMC by contributions. In these omics datasets, the contributions of the first level were significantly greater than those of the second (Supplementary Fig. S9). This suggests that eigen-phases 1A and 1B capture the primary molecular variability within the YMC, whereas eigen-phases 2A and 2B play secondary roles.

### Interplay of induction and repression of four eigen-phases

Another insight is that the four eigen-phases demonstrate an interplay of induction and repression (Fig. 2G).

First, the adjacent eigen-phases overlap, indicating an inductive relationship. This overlap suggests that the transition between eigen-phases is not abrupt but rather governed by a gradual progression (Fig. 2A–E). In this progression, each phase plays roles of both the outcome of the preceding one and the initiator of the next. Thus, we proposed that each eigen-phase induces the next one.

Second, there is reciprocal repression between eigen-phases at the same level. Specifically, eigen-phases 1A and 1B, as well as 2A and 2B, reciprocally repressed each other. This reciprocal repression is manifested by the opposite signs of sample loadings (Fig. 2A–E).

### Consistency of eigen-phases across different omics datasets

The four eigen-phases were separately defined within the transcriptome, epigenome (for H3K9ac, H3K18ac, H3K56ac, and H4K5ac), and metabolome, raising questions about their consistency across different omics. To address this, we analyzed their sample- and molecule-eigenvectors at the top two levels.

To analyze sample-eigenvectors, we first aligned samples from different datasets based on dissolved oxygen concentration (Supplementary Fig. S7), as it is a key and stable indicator of metabolic activity in the YMC. Specifically, the different oxygen curves were aligned by the DDTW method [25]. Based on the DDTW results, samples from different datasets were aligned on a unified timeline. Next, we calculated the maximum cross-correlation, a measure of temporal consistency, of sample-eigenvectors between the transcriptome and other datasets (“Materials and methods” section). This analysis revealed high maximum cross-correlation values (>0.8) between transcriptome and epigenome for H3K9ac, H3K18ac, and H4K5ac (Supplementary Table S2). In comparison, the average maximum cross-correlation across two levels between transcriptome and metabolome was slightly lower, at 0.796. These results indicate the temporal consistency of sample-eigenvectors between these datasets.

The molecule-eigenvector represents genes in both transcriptome and epigenome datasets. Thus, we calculated their pairwise correlation coefficients (Supplementary Fig. S11). This analysis showed that signals of genes regulated by H3K9ac and H3K18ac exhibited higher correlation coefficients (>0.5) with expression levels, indicating a consistency between these datasets. Furthermore, gene enrichment and key metabolites analyses, to be elaborated in subsequent sections, further supported the consistency between the transcriptome and metabolome.

These findings confirmed the consistency of these four eigen-phases between transcriptome and other datasets including epigenome for H3K9ac, H3K18ac, and metabolome. In the following analysis, we focused mainly on these datasets.

### Representation of molecular cyclic signals

During the YMC, many molecules exhibited cyclic signals such as gene expression levels and metabolite concentrations [3, 6, 7]. By focusing on SVD at the scale of individual molecules, we obtained a mathematical representation of these molecular cyclic signals (Fig. 1E). Each molecular cyclic signal, as a function of time and molecule, was decomposed into a baseline, two levels, and other factors. Each level was expressed as the product of a singular value, a molecule loading function, and a periodic function. The periodic function captures the temporal dynamic of the molecular signal during the YMC, whereas the singular value and molecule loading together quantify the variance of the signal at each level. Notably, the singular value quantifies the overall variance across all molecules at each level. The higher singular value at level 1 indicates greater molecular variability compared to level 2.

### Timing of multi-omics dynamics among eigen-phases

**Figure 3.**
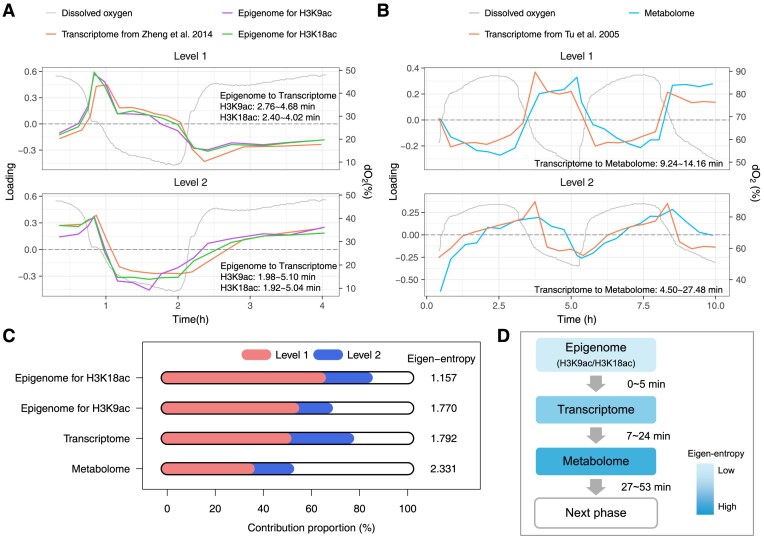
(**A** and **B**) Comparison of sample loadings across different omics datasets on a unified timeline at the top two levels. Samples from different omics datasets are aligned by dissolved oxygen concentrations using DDTW. Different datasets exhibit consistent YMC phase patterns. Furthermore, this analysis reveals a temporal order of the epigenome (for H3K9ac/H3K18ac), transcriptome, and metabolome in a single phase. Time differences between these datasets are estimated using the shift parameter in the periodic functions (Fig. 1F), with results marked alongside the curves. (**C**) Relative contributions of the top two levels in different omics datasets. Relative contributions are calculated as the ratio of the squared singular value of each level to the total sum. The eigen-entropy is defined as the entropy of a multinomial distribution constructed by the relative contributions across all levels. (**D**) A model of dynamic multi-omics across each eigen-phase. The shade gradient from light to dark represents increasing eigen-entropy, following the temporal order of the epigenome (for H3K9ac/H3K18ac), transcriptome, and metabolome. The estimated time differences are marked on the right.

Notably, after plotted on a unified timeline, different omics datasets were found following a temporal progression: starting with the epigenome (for H3K9ac/H3K18ac), followed by the transcriptome, and then the metabolome (Fig. 3A and B). The time differences between omics data were represented by the shift parameter in the periodic functions (Fig. 1F). To estimate this time difference, we computed the time lag corresponding to the maximum cross-correlation between two sample-eigenvectors (“Materials and methods” section). We further inferred the confidence intervals of time differences through a method based on perturbation. These results are presented in Supplementary Table S3.

This analysis revealed that the time differences from epigenome (for H3K9ac/H3K18ac) to transcriptome at the top two levels averaged ∼3 min, with 95% confidence intervals ranging from 1.92 to 5.04 min. In contrast, the time differences from transcriptome to metabolome averaged ∼13 min, with 95% confidence intervals ranging from 4.50 to 27.48 min.

These findings supplement previous observations of time differences between specific epigenomic modifications and certain gene clusters [7]. Notably, the minimal time lags are from epigenome, H3K9ac and H3K18ac, to transcriptome. Since the transcriptome reflects spliced mature mRNA, this minimal lag corresponds to partial mRNA splicing time, which has been found to average 7.3 min in certain genes [7].

We also estimated the time differences from a metabolomic eigen-phase to the next transcriptomic one (Supplementary Table S3). The results showed that this time difference was ∼38 min, with 95% confidence intervals ranging from 29.64 to 53.7 min.

### Eigen-entropy increases from epigenome H3K9ac/H3K18ac to metabolome

The total relative contributions of the top two levels, quantified by the ratios of squared singular values, varied significantly across different omics datasets (Fig. 3C). To further investigate this variation, we calculated the eigen-entropy for each dataset (Fig. 3C). Here, eigen-entropy is defined as the entropy of a multinomial distribution, derived from the ratios of squared singular values across all levels. Along the temporal order from epigenome H3K9ac/H3K18ac to metabolome, our analysis showed an increase in eigen-entropy (Fig. 3D), indicating the growing complexity of biological processes from transcriptional regulation to metabolic activity over time.

### Signature pathways and marker genes of each eigen-phase

**Figure 4.**
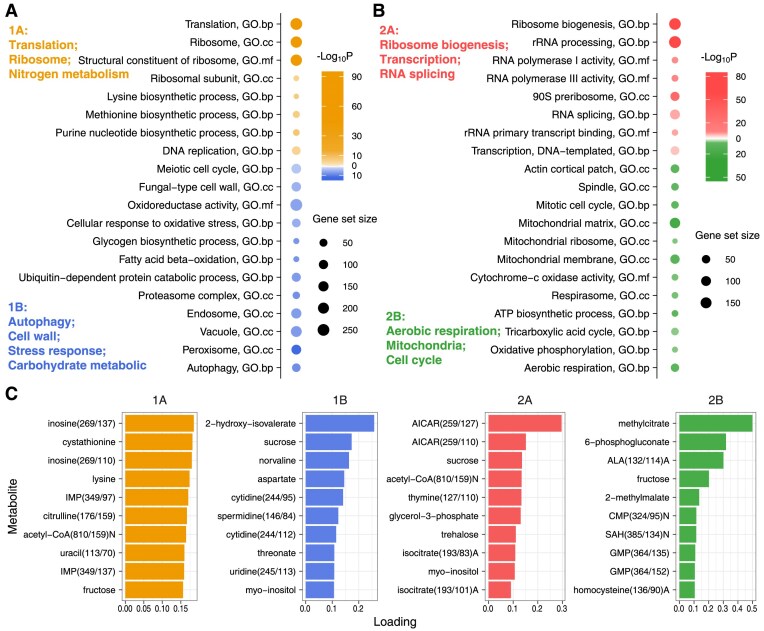
(**A**) Representative pathways that significantly enriched in the two eigen-phases at the first level. The bold text on the left summarizes key biological processes associated with these pathways. Top, eigen-phase 1A; bottom, eigen-phase 1B. (**B**) Representative pathways that significantly enriched in the two eigen-phases at the second level. Top, eigen-phase 2A; bottom, eigen-phase 2B. (**C**) Key metabolites in each eigen-phase. Ten metabolites with the highest loadings in each eigen-phase are shown.

To investigate the signature biological processes of each eigen-phase, we performed enrichment analysis on gene-eigenvectors of the transcriptome, identifying up-regulated pathways. A representative subset of these signature pathways is illustrated in Fig. 4A and B. These pathways were selected based on the following criteria: first, pathways that are most statistically enriched were chosen. Second, among pathways with similar biological descriptions, a single representative one was selected. Third, pathways statistically significant in two adjacent eigen-phases were assigned to the earlier one.

In summary, eigen-phase 1A was characterized by translation and amino acid metabolism; 1B by degradation mechanisms and stress responses; 2A by ribosome biogenesis; and 2B by aerobic respiration, mitochondria, and cell cycle. Further details of these signature pathways and their implications are available in the Supplementary Text.

Furthermore, each eigen-phase is potentially marked by specific marker genes. To identify these critical marker genes, we employed a novel statistical method involving the randomization of sample-eigenvectors. This method revealed significant marker genes (Supplementary Fig. S12), which were closely associated with the signature biological processes of each eigen-phase. Further details regarding these marker genes are available in Supplementary Tables S4–S7.

### Opposite regulation of ribosome biogenesis/aerobic respiration between eigen-phases 2A/2B

In transcriptome, the reciprocal repression of eigen-phases at the same level was manifested by the up-regulation of specific pathways in one eigen-phase, coupled with their down-regulation in the opposite eigen-phase. In particular, the opposite regulation between ribosome biogenesis in eigen-phase 2A and aerobic respiration in 2B has been recognized in multiple studies [13, 35, 36]. These examples underscore the pervasive nature of reciprocal repression, not only within the YMC but also throughout yeast biology.

### Key metabolites of each eigen-phase

Key metabolites in each eigen-phase were identified based on their high loadings in metabolite-eigenvectors of metabolome (Fig. 4C). These metabolites exhibited strong correlations with the signature biological processes of each eigen-phase. For example, spermidine, which had high loading in eigen-phase 1B, is known to promote longevity, inhibit oxidative stress, suppress necrosis, and enhance autophagy [37]. In eigen-phase 2A, 5-aminoimidazole-4-carboxamide ribonucleotide (AICAR) can stimulate the activity of AMP-dependent protein kinase (AMPK), which enhances glucose uptake and oxidation for energy production [38]. Another notable metabolite is acetyl-CoA, a key intermediate in the tricarboxylic acid cycle (TCA cycle), where it initiates reactions producing ATP, NADH, and FADH2, essential for cellular energy. Its high loading in both eigen-phases 1A and 2A indicates a high-energy state in the cell. Further details regarding key metabolites and their implications are available in the Supplementary Text.

### Statistical inference of regulatory transcription factors at each eigen-phase

**Figure 5.**
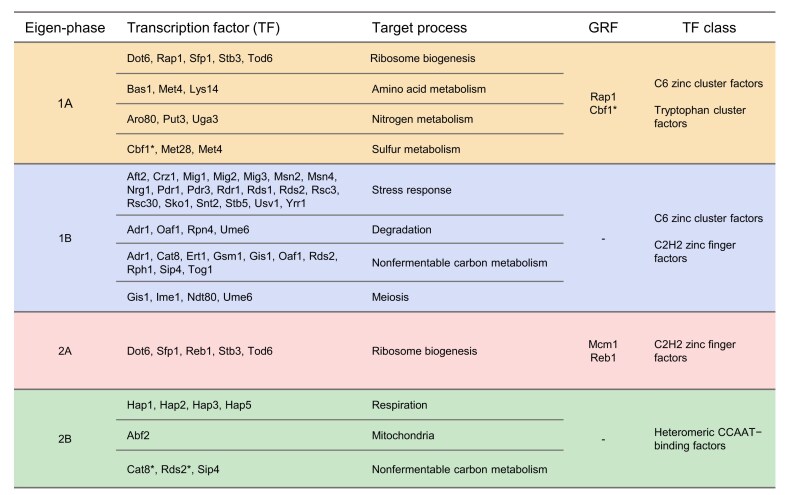
Summary of the regulation by TFs in each eigen-phase. Significant TFs regulating each eigen-phase are inferred using BASE2.0. TFs are grouped by their target biological processes. These TFs include several GRFs. These TFs are also associated with specific classes. *TFs are the significant factors identified in the BASE2.0 analysis of data from [1] alone.

To investigate transcriptional regulation mechanisms in each eigen-phase, we employed the BASE2.0 method to identify significant TFs for each transcriptomic eigen-phase (Supplementary Tables S8–S11) [28]. Our analysis revealed a strong correlation between these TFs and the signature biological processes of each eigen-phase (Fig. 5). For example, Msn2p and Msn4p, significant in eigen-phase 1B, are pivotal regulators of the general stress response [39], aligning with the stress response processes of this eigen-phase. A comprehensive discussion of these significant TFs is available in the Supplementary Text.

Notably, four out of the five general regulatory factors (GRFs) were identified as significant (Fig. 5). GRFs are known not only for their extensive binding sites across the genome but also for enhancing chromosomal accessibility and the activity of proximal TFs [40–42]. This finding suggests a potential link between GRFs and the regulatory mechanisms driving the YMC eigen-phases.

TFs can be classified based on their DNA-binding domains, showing their structural and functional diversity [43]. Utilizing TF classifications from the JASPAR database [30], we identified statistically significant associations between TF classifications and specific eigen-phases by contingency table tests (Supplementary Fig. S13). This analysis revealed that C6 zinc cluster factors are pivotal in eigen-phases 1A and 1B, whereas C2H2 zinc finger factors are prominent in eigen-phases 1B and 2A. These findings underscore the critical role of zinc cluster proteins in YMC regulation [44]. Notably, C6 zinc cluster proteins are unique to fungi [45], suggesting specialized regulatory mechanisms within the YMC.

### Difference between histone acetylation H3K9ac/H3K18ac and methylation H3K4me3

The sample loading curves of the epigenome H3K9ac/H3K18ac and transcriptome revealed a highly similar pattern (Fig. 3A). Both the cross-correlation of their sample-eigenvectors and the correlation of their gene-eigenvectors confirm relatively high consistency between these datasets (Supplementary Fig. S11 and Supplementary Table S2). Besides, the top two levels contributed significantly (>60%) in all these datasets (Supplementary Fig. S9). The results suggest that H3K9ac and H3K18ac have a general regulatory impact on gene expression throughout the YMC, in line with previous studies that indeed identified the modifications as key drivers of the YMC [9].

To further investigate the roles of histone modifications, we calculated their relative contributions across the four transcriptomic eigen-phases. The calculation involved several steps. First, a transcriptomic gene-eigenvector ${u_1}$ was decomposed into positive and negative components $u_1^ +$ and $u_1^ -$, such that ${u_1} = u_1^ + - u_1^ -$, where their elements were defined as $u_{1i}^ + = \max \{ {{u_{1i}},0} \},\;u_{1i}^ - = \max \{ { - {u_{1i}},0} \}$. After normalizing them so that their elements sum to 1, $u_1^ + \;$and $u_1^ -$ represented the gene weights of eigen-phases 1A and 1B, respectively. Similarly, the dual transcriptomic sample-eigenvector ${v_1}$ was decomposed into $v_1^ + \;$and $v_1^ -$, representing the sample weights of eigen-phases 1A and 1B, respectively. Next, for a given histone modification, its signal matrix ${A^{( l )}}$ was interpolated to match the transcriptomic sampling time points. Finally, the contributions of this histone modification to eigen-phases 1A and 1B were calculated as $u_1^+{} ^T{A^{( l )}}v_1^ +$ and $u_1^-{}^T{A^{( l )}}v_1^ -$, respectively. Contributions to eigen-phases 2A and 2B were calculated similarly as $u_2^+{}^T{A^{( l )}}v_2^ +$ and $u_2^ -{} ^T{A^{( l )}}v_2^-$.

The results are shown in Supplementary Fig. S14. Due to incomplete data, H3K36me3 and H4K16ac were not included in the analysis. For each histone modification, the coefficient of variance (CV) was also calculated to evaluate the uniformity of its contribution across eigen-phases. A lower CV represents a more even contribution. The results show a uniform contribution of H3K9ac/H3K18ac across all eigen-phases, with the CV < 0.2. In contrast, the histone methylation H3K4me3 shows a higher CV of 0.5, indicating it makes phase-specific contributions. This phase-specific pattern is in line with changes observed in the metabolome. In eigen-phase 2B, where H3K4me3 showed the highest contribution, we observed elevated levels of S-adenosylmethionine (SAM), the primary methyl donor (Supplementary Fig. S15). This abundance of SAM suggests an increased capacity for histone methylation activities, particularly for the H3K4me3 [46, 47].

These findings indicate different roles of histone acetylation and methylation in transcriptional regulation during the YMC. While H3K9ac and H3K18ac ensure transcriptional stability and continuity throughout the YMC, H3K4me3 plays a phase-specific role. This difference reflects a cellular strategy in maintaining both robustness and adaptability in a dynamic metabolic environment. Further investigation is required to elucidate the relationship between these modifications and their broader cellular functions.

### Production/consumption of glycerol in eigen-phases 2A/2B

**Figure 6.**
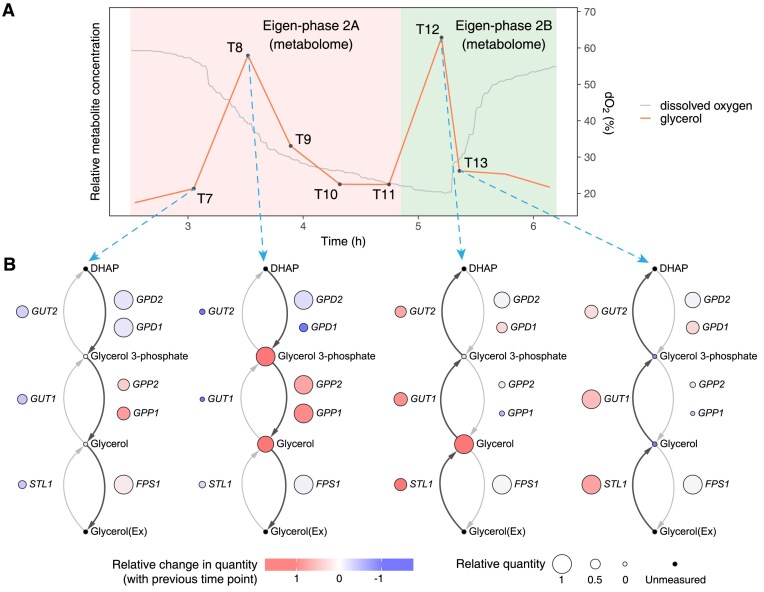
(**A**) The relative concentration of glycerol from time points T7 to T13. It exhibits a dual peak pattern in a single cycle. The two peaks occur in metabolomic eigen-phases 2A and 2B, respectively. (**B**) Glycerolipid metabolic network during the period of the two glycerol concentration peaks. This indicates a metabolic shift of glycerol from production to consumption during this period. DHAP, dihydroxyacetone phosphate; Ex, extracellular.

Glycerol is a fascinating metabolite that displayed a dual concentration peak pattern in one cycle (Fig. 6A). These two peaks occurred in eigen-phases 2A and 2B, respectively. To further investigate this phenomenon, we constructed a detailed glycerolipid metabolic network around these two peaks (Fig. 6B).

At the first peak in eigen-phase 2A, our analysis revealed a metabolic flux from glycerol 3-phosphate to glycerol, catalyzed by the enzymes Gdp2, Gpp1, and Gpp2 [48, 49]. The produced glycerol was then exported to the extracellular matrix, regulated by the Fps1 transporter [50].

At the second peak in eigen-phase 2B, glycerol underwent a reverse metabolic pathway. It was transported from the extracellular matrix to the intracellular one. This reabsorption process was facilitated by the high expression level of the Stl1 transporter [51]. The reabsorbed glycerol was then converted back to glycerol 3-phosphate, driven by the enzymes Gut1 and Gut2 [52, 53].

Glycerol serves as a by-product under fermentation to consume excess NADH and is also used as the carbon source when lack of nutrients [54, 55]. Therefore, the cyclic production and consumption of glycerol during the YMC suggest different nutrient availability and metabolic status in eigen-phases 2A and 2B.

## Discussion

**Figure 7.**
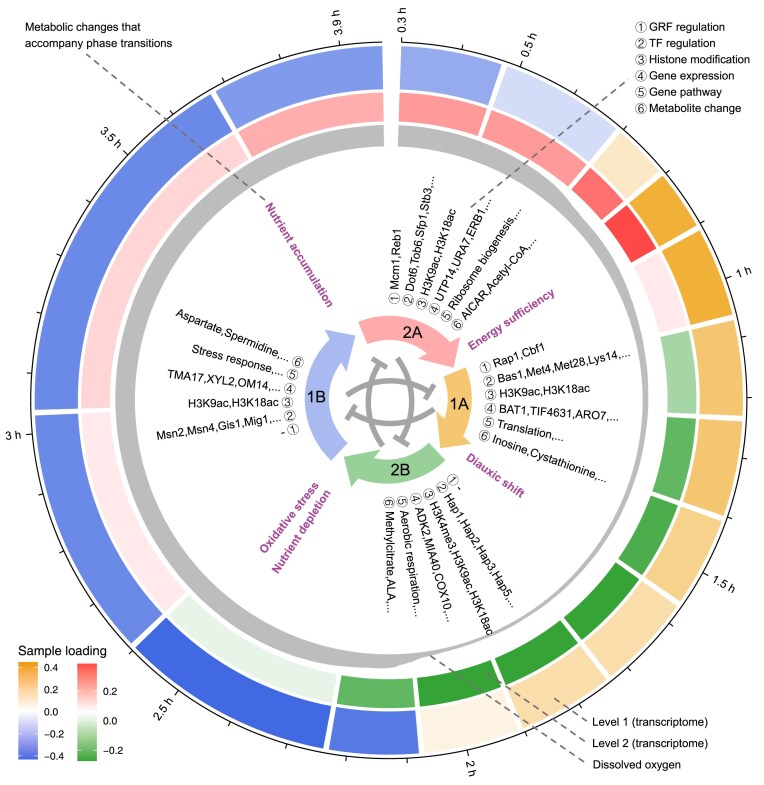
The unified landscape of the YMC. The outermost layer depicts the timeline. In the outer layer, sample loadings of the transcriptome at the top two levels and dissolved oxygen concentrations are shown. The middle layer shows key molecules and pathways associated with each eigen-phase, including GRFs, TFs, histone modifications, marker genes, gene pathways, and key metabolites. This layer also shows metabolic changes that accompany eigen-phase transitions. In the inner layer, the four eigen-phases are arranged in a circular sequence, indicating the induction from one eigen-phase to the next. Reciprocal repression between eigen-phases at the same level is depicted in the center.

Using the proposed SVD-based synthesis approach, we have elucidated a four eigen-phase YMC model that demonstrates an interplay of induction and repression. Each eigen-phase is characterized by its signature gene expressions, transcriptional regulation mechanisms, and metabolite changes. Figure 7 summarizes these findings by a unified landscape of the YMC.

### The four eigen-phase model

We established a correspondence between the traditional YMC phases (OX, RB, and RC) and the new eigen-ones by a thorough comparison of their enriched biological processes. At the mRNA expression level [3], eigen-phase 1A and 2A together align closely with OX, 1B with RC, and 2B with RB. Further comparison revealed that eigen-phases 2A and 1A respectively correspond to OX1 and OX2, subclusters of the OX phase [7]. The OX1 mainly contains genes related to ribosome biogenesis (ribi) and rRNA processing, while OX2 genes are involved in ribosome protein (RP), amino acid metabolism, and translation.

The new four eigen-phase model subdivides the traditional OX phase into eigen-phases 1A and 2A. This division is not a mathematical artifact but rather a biologically meaningful model for the YMC. Here, we offer several perspectives to support this model. First, ribi genes in 2A were up-regulated immediately before the expression of RP and translational genes in 1A, suggesting a potential just-in-time coordination mechanism essential for enhancing translational capacity during the YMC [7]. It is noted that the transition from 2A to 1A occurs when the oxygen level decreases sharply.

Second, in the transcriptome, epigenome for H3K9ac/H3K18ac, and metabolome, the contributions to the total variance of data in 1A/1B at the first level was significantly greater than that at the second one (Supplementary Fig. S9), indicating biological processes in eigen-phase 1A are more active and energy-consuming than those in 2A.

Third, the two eigen-phases at the same level are reciprocally repressed as indicated by their positive and negative sample loadings (Fig. 7, central part). This reciprocal repression, or opposite regulation, also recognized in multiple studies [13, 35, 36], indicates a critical aspect of cellular homeostasis and adaptability: molecular processes activated in one eigen-phase are repressed in the opposite eigen-phase. For example, eigen-phases 2A and 2B are characterized respectively by rRNA biogenesis and aerobic respiration. Interestingly, we reported their opposite regulation in the long-lived mutants *sch9Δ* [35], which has a threefold chronological lifespan in the nutrition-limited SDC medium compared to the wild-type strain DBY746. The opposite regulation of rRNA biogenesis and aerobic respiration is believed to be a mechanism that protects genome stability. This mechanism is also observed in the metabolic cycle of the wild-type strain CEN.PK122.

In contrast to CEN.PK122, common laboratory strains S288C and W303 do not exhibit the YMC [3]. This observation suggests that the YMC is a critical adaptive mechanism of wild yeasts for survival in the natural environment. Through the interplay of induction and repression between eigen-phases, cells manage to survive by optimizing the use of energy and oxygen while maintaining genome integrity.

### Metabolic changes accompanying eigen-phase transitions

The transition between eigen-phases is an interesting aspect of the YMC. To find cues about dynamics in eigen-phase transition, we plotted the central carbon metabolism at four selected time points that represent the four eigen-phases respectively (Supplementary Fig. S16). We observed the following metabolic changes accompanying eigen-phase transitions.

Nutrient accumulation precedes the transition from eigen-phase 1B to 2A. Specifically, we observed the up-regulation of glycogen and trehalose biosynthesis pathways in eigen-phase 1B (Fig. 4A), and the elevated concentrations of carbohydrate storage-related metabolites (Supplementary Fig. S16A). Moreover, previous studies showed that the duration of the reductive eigen-phase 1B in YMC increases as available glucose decreases [56, 57], indicating a relationship between glucose accumulation and eigen-phase timing.

The transition from eigen-phase 2A to 1A is accompanied by energy sufficiency. The elevated glycolysis, TCA cycle, fermentation, and glycerolipid metabolism in eigen-phase 2A indicate enhanced energy production (Supplementary Fig. S16B). During 1A, the key metabolite acetyl-CoA, maintained at a high level, further indicates a high-energy status [7, 58] (Fig. 4C and Supplementary Fig. S17). Eigen-phase 1A is characterized by intensive translational activities (Fig. 4A), suggesting that the energy surplus from 2A fuels these activities.

We observed the diauxic shift from eigen-phase 1A to 2B. Diauxic shift is the metabolic reprogramming from anaerobic fermentation (glucose utilization) to aerobic respiration (ethanol utilization) [36, 59]. Both the reduction in glucose levels and the increase in extracellular ethanol concentrations indicate anaerobic fermentation during 1A (Supplementary Fig. S18). After entering eigen-phase 2B, extracellular ethanol concentrations dropped, indicating cells adaptively respond to glucose depletion by metabolizing available ethanol. Further evidence for the diauxic shift is provided by transcriptome analysis, which revealed an up-regulated TCA cycle and concurrent down-regulated translation from eigen-phase 1A to 2B (Fig. 4A and B), in line with the transcriptional markers of the diauxic shift [36]. Moreover, the metabolome revealed a metabolic shift from glycolysis to gluconeogenesis during the transition from eigen-phase 1A to 2B (Supplementary Figs S16C and D), consistent with metabolite changes observed during the diauxic shift [59].

Oxidative stress and nutrient depletion preceded the transition from eigen-phase 2B to 1B. 1B is characterized by the up-regulation of stress response (Fig. 4A). Concurrently, the depletion of nutrients in late 2B likely contributes to the transition. The metabolic signature in 1B, to some extent, resembles that observed in the yeast stationary phase, which is characterized by enhanced stress response, autophagy, and carbohydrate storage, as well as reduced translation [60–62]. Generally, cells transition to the stationary phase when the carbon source is depleted. In addition, meiosis in yeast is also up-regulated under starvation conditions [63].

### Energy homeostasis during YMC

Energy homeostasis is a critical aspect of the YMC. Previous studies proposed a feedback system in YMC, demonstrating that the energy state, reflected by the ATP: ADP ratio, regulated gene transcription [13]. This energy state is also reflected by the cyclic levels of acetyl-CoA [7, 58]. In this study, we identified more key metabolites related to energy homeostasis. AICAR senses energy changes and activates AMPK when AMP: ATP ratio decreases, promoting the metabolic pathways related to energy production [38]. It was important in eigen-phase 2A (Fig. 4C), suggesting its key role in energy production during this eigen-phase to initiate a new cycle. Another metabolite, glycerol, exhibits cyclic production and consumption, indicating the storage and usage of an alternative energy source (Fig. 6B), also reflecting the energy state of different YMC eigen-phases.

### Timing and entropy in YMC biological processes

The timing of different omics is an important finding (Fig. 3A and B), which could not be discovered without integrating data from various sources. While previous studies have investigated the relative timing of histone modifications and RNA signals for several gene clusters [7], this study provides a systematic estimation of the timing across the YMC. The global timing reflects a general molecular regulatory mechanism: histone acetylation marks, especially H3K9ac and H3K18ac, act as earlier regulatory signals for transcription. The temporal precedence of epigenetic modifications may provide adaptive advantages, as chromatin modifications could respond to metabolic and environmental cues, such as acetyl-CoA levels [58]. The time lag from epigenome H3K9ac/H3K18ac to transcriptome, and subsequently to metabolome would help build mathematical dynamic equations of YMC in future research.

The increase in eigen-entropy from epigenome H3K9ac/H3K18ac to metabolome was observed (Fig. 3D). Heuristically, tightly regulated molecular signals are characterized by larger differences in singular values across levels, resulting in lower eigen-entropy. The observed pattern may reflect the entropy-increasing nature from transcription to translation: epigenetic modifications H3K9ac/H3K18ac, act as earlier signals in quick response to available nutrition, tightly coordinated with other factors to regulate gene transcription. Post-transcriptional modification, such as alternative splicing and RNA editing, may increase entropy by generating a greater diversity of biomolecules [64]. Furthermore, metabolomic processes, being more dynamic and diverse, reflect increasingly complex cellular responses and interactions. The eigen-entropy provides a novel computational perspective for analyzing the dynamics of biological systems.

As more omics data of the YMC become available, they can be integrated with existing ones through our synthesis approach. The SVD-based dual eigen-analysis and alignment of datasets is a general framework applicable to other data integration problems.

## Supplementary Material

lqaf022_Supplemental_File

## Data Availability

The data underlying this article are available in the article and its online supplementary material. The source codes are available at https://doi.org/10.5281/zenodo.14671612
