# Supplementary material for: A four eigen-phase model of multi-omics unveils new insights into yeast metabolic cycle
Source: NAR Genom Bioinform. 2025 Mar 19;7(1):lqaf022. doi: 10.1093/nargab/lqaf022 (PMC11920873; doi:10.1093/nargab/lqaf022)
Supplement: lqaf022_Supplemental_File [file lqaf022_supplemental_file.pdf]

Supplementary Material

A Four Eigen-Phase Model of Multi-Omics Unveils New Insights into the Yeast Metabolic Cycle

Linting Wang[1,2], Xiaojie Li[1,2], Jianhui Shi[1,2], Lei M. Li[1,2,*]

[1]National Center of Mathematics and Interdisciplinary Sciences, Academy of Mathematics and Systems Science, Chinese Academy of Sciences, Beijing, China

[2]University of the Chinese Academy of Sciences, Beijing, China

*Corresponding author. Email: lilei@amss.ac.cn

Contents

Supplementary Text 1
The evaluation of data integration methods 1
Transcriptome concatenation method 1
Metabolome alignment method 1
The up-regulated pathways of each eigen-phase 2
Eigen-phase 1A: Translation 2
Eigen-phase 1B: Degradation mechanisms and stress response 2
Eigen-phase 2A: Ribosome biogenesis 3
Eigen-phase 2B: Aerobic respiration 3
The roles of significant transcription factors in regulating each eigen-phase 3
Eigen-phase 1A 3
Eigen-phase 1B 4
Eigen-phase 2A 5
Eigen-phase 2B 5
The roles of key metabolites of each eigen-phase 5
Eigen-phase 1A 5
Eigen-phase 1B 6
Eigen-phase 2A 7
Eigen-phase 2B 7
Supplementary Figures 9
Figure S1. The schematic representation of dual eigen-analysis. 9
Figure S2. Densities of pairwise differences between samples T1 and T7 of epigenome H3K9ac before and after normalization by MUREN. 10
Figure S3. Loadings of the 0-th sample-eigenvector of omics data. 11
Figure S4. The preprocessing and concatenation of two expression profile matrices. 12
Figure S5. Densities of two transcriptomics datasets before and after scaled by F-norm. 12
Figure S6. Oxygen concentration curves of each omics data. 13
Figure S7. Oxygen concentration curves of different omics data after DDTW alignment 14
Figure S8. Loadings of the sample-eigenvectors at the top two levels in

transcriptome and metabolome datasets. 15
Figure S9. Percentages of squared singular values of the top five eigen-components of omics data..16
Figure S10. Loadings of the sample-eigenvectors at the top two levels in epigenome datasets for H3K56ac and H4K5ac. 17
Figure S11. Pairwise Pearson correlation coefficients between gene-eigenvectors of transcriptome and epigenome at the top two levels.......................................17
Figure S12. Volcano plots of the marker genes in each eigen-phase...................18
Figure S13. Counts for classifications of significant TFs in each eigen-phase. ..18
Figure S14. Relative contribution of histone modifications to each eigen-phase19
Figure S15. Relative concentration of S-adenosylmethionine (SAM). 19
Figure S16. Central carbon metabolism network of four eigen-phases..............20
Figure S17. Relative concentration of acetyl-CoA. 21
Figure S18. Relative concentration of ethanol (extracellular) and glucose. 21
Supplementary Tables 23
Table S1. Summary of YMC omics datasets. 23
Table S2. Maximum cross-correlation of sample-eigenvectors between transcriptome and other omics datasets at the top two level...............................23
Table S3. Time differences between different omics datasets. 24
Table S4. Marker genes of eigen-phase 1A. 24
Table S5. Marker genes of eigen-phase 1B. 25
Table S6. Marker genes of eigen-phase 2A. 27
Table S7. Marker genes of eigen-phase 2B. 29
Table S8. Significant TFs of eigen-phase 1A. 33
Table S9. Significant TFs of eigen-phase 1B...34
Table S10. Significant TFs of eigen-phase 2A. 36
Table S11. Significant TFs of eigen-phase 2B...37
References 39

Supplementary Text

The evaluation of data integration methods

Transcriptome concatenation method

The transcriptomic data matrices of yeast metabolic cycle (YMC) expression profiles were sourced from two studies (1,2), utilizing microarray and RNA-seq techniques. Subsequently, we integrated these two matrices into a unified matrix through concatenation [Figure S4]. This concatenation method was proposed and evaluated in our earlier study (3).

To evaluate the effectiveness of our concatenation method, we compared the singular value decomposition (SVD) results of the concatenated matrix with those of each original dataset. First, the analysis revealed that the singular values at the top two levels of each original dataset contribute significantly, mirroring those of the concatenated matrix [Figure S9A]. Second, the correlation coefficients of gene-eigenvectors between the concatenated matrix and the original datasets were high—0.96 and 0.97 for the first level, as well as 0.96 and 0.95 for the second [Figure S11]. Third, the four eigen-phases were consistently identified in each original dataset, aligning with those of the concatenated matrix [Figures S8A and S8B]. These results collectively demonstrated the effectiveness of our concatenation method in capturing the essence of the original data.

In addition, the top two gene-eigenvectors of the two original datasets both exhibit a high correlation coefficient of 0.85 [Figure S11]. This pronounced correlation indicates that the top two eigen-components are conserved across the datasets, suggesting that they capture fundamental and stable patterns of gene expression relevant to the YMC.

Metabolome alignment method

The metabolite concentrations in the metabolome data were measured using two methods, Liquid chromatography tandem mass spectrometry (LC-MS) and comprehensive 2D gas chromatography time-of-flight mass spectrometry (GC-TOFMS), at different sampling time points (4). To obtain an integrative result, we aligned them through linear interpolation, mapping the metabolite data from GC-TOFMS to those from LC-MS. To evaluate the effectiveness of the alignment method, we compared its SVD result to those of each original dataset and another aligned dataset, which was obtained by mapping the metabolite data from LC-MS to those

from GC-TOFMS.

The analysis revealed that the patterns of singular values in these data matrices were highly similar, as they all showed the importance of the top two levels [Figure S9B]. In addition, the four eigen-phases were also consistently identified in each data matrix [Figures S8C-E]. These results collectively demonstrated the effectiveness of our alignment method.

The up-regulated pathways of each eigen-phase

Eigen-phase 1A: Translation

Eigen-phase 1A was characterized by an up-regulation of translation and its associated pathways, underscoring translation as the primary biological process in this eigen-phase [Figure 4A].

This enhancement was evident not only in the up-regulation of translation pathways directly but also in the concurrent elevation of related pathways, including those involving the ribosome—the site of protein translation—and amino acid metabolism. These interrelated up-regulations suggest a comprehensive cellular strategy aimed at boosting protein synthesis by ensuring an ample supply of amino acids.

Eigen-phase 1B: Degradation mechanisms and stress response

In eigen-phase 1B, the up-regulated pathways were predominantly associated with degradation mechanisms and stress responses [Figure 4A].

Notably, autophagy and the ubiquitin-proteasome system, both critical for intracellular degradation and recycling (5), were markedly enhanced. Autophagy eliminates unnecessary or damaged cellular components through a highly regulated self-degradative process (6), while the ubiquitin-proteasome system tags defective proteins for degradation, underscoring the cell's prioritization of maintaining homeostasis under stress (7).

Furthermore, we identified a concurrent up-regulation in pathways associated with the cellular response to oxidative stress, along with mechanisms for carbohydrate storage, cell wall formation, and meiosis. This comprehensive up-regulation suggests a strategic cellular adaptation to stress by reinforcing processes that are essential for survival, degradation, and recycling.

These findings collectively revealed the adaptive response of yeast during eigen-phase 1B, mitigating stress impacts through enhanced degradation and recycling pathways.

This underscores the intricate relationship between stress responses and cellular degradation mechanisms, illustrating their crucial roles in maintaining cellular integrity under adverse conditions.

Eigen-phase 2A: Ribosome biogenesis

Eigen-phase 2A is characterized by a predominant up-regulation of pathways related to ribosome biogenesis [Figure 4B]. This eigen-phase exhibited a comprehensive enhancement in mechanisms essential for ribosome assembly, including the transcription, export, splicing, and processing of rRNA. This concerted up-regulation underscores the cell's strategic adaptation for protein synthesis, revealing a preparatory step for translation.

Eigen-phase 2B: Aerobic respiration

In eigen-phase 2B, the up-regulated pathways were primarily associated with aerobic respiration [Figure 4B].

This eigen-phase was characterized by a significant up-regulation of pathways central to aerobic respiration, a critical energy-producing process within the mitochondria that involves the tricarboxylic acid (TCA) cycle and oxidative phosphorylation, culminating in ATP production. Specifically, the pathways related to the TCA cycle, oxidative phosphorylation, and ATP biosynthesis were notably elevated, reflecting the optimizing cellular energy production.

Moreover, this eigen-phase exhibited an up-regulation in pathways critical for maintaining and enhancing mitochondrial function, including mitochondrial matrix, mitochondrial ribosomes, and mitochondrial membrane, further underscoring the cell's commitment to efficient energy production. Additionally, the up-regulated cell cycle and related pathways indicate a link between increased energy availability and cellular proliferation.

These findings collectively highlight the central theme of eigen-phase 2B: a focused enhancement of aerobic respiration, alongside concerted efforts in mitochondrial biogenesis and cell cycle activation.

The roles of significant transcription factors in regulating each eigen-phase

Eigen-phase 1A

In eigen-phase 1A, the transcription factors (TFs) were primarily associated with ribosome assembly and metabolic regulation. Dot6p and Tod6p are known for their association with PAC-binding proteins, while Stb3p is with the RRPE motif (8,9). The significance of these TFs reveals a complex regulatory network crucial for processes including ribosome assembly, transcription, and translation, which are integral to rapid cellular growth. In addition, Sfb1p is a key regulator of ribosomal protein gene expression in response to both nutrient availability and stress conditions (10), and Rap1p extensively regulates ribosomal protein genes (11).

Two general regulatory factors (GRFs), Rap1p and Cbf1p, are present in this eigen-phase. Rap1p, exhibiting characteristics similar to mammalian pioneer factors, activates a wide array of genes, including encoding for ribosomal proteins (11-13). This regulatory role aligns with the enhanced ribosome activity observed in eigen-phase 1B [Figure 4A]. Cbf1p is involved in the regulation of sulfur metabolism (14), a pathway that is prominently up-regulated in eigen-phase 1B [Figure 4A].

Furthermore, TFs such as Lsy14p, Met4p, and Bas1p are specialized in the biosynthesis of specific amino acids (15-17). Met4p and Met28p play key roles in the regulation of sulfur metabolism (18), and Put3p, Uga3p, and Aro80p exert regulatory control over nitrogen metabolism (19-21).

Eigen-phase 1B

In eigen-phase 1B, a substantial number of TFs play critical roles in responding to various stress conditions. Notably, Msn2p and Msn4p act as pivotal regulators of general stress response (22). Furthermore, TFs Aft2p, Nrg1, Sko1p, Snt2p, and Stb5p are crucial in responding to oxidative stress (23-27), while a group of regulators, including Pdr1p, Pdr3p, Rdr1p, Rds1p, Rds2p, Stb5p, and Yrr1p, are involved in controlling multidrug resistance (28-31). Eigen-phase 1B also involves TFs like Mig1p and Mig2p, which are pivotal in responding to amino acid starvation (32); Mig3p, which addresses genotoxic stress (33); Usv1p and Sko1p, which are involved in salt/osmotic stress adaptation (25,34); Crz1p, which responds to blue light stress (35); Rsc3p and Rsc30p, which play roles in a broader spectrum of stress responses (36).

The significant TFs in eigen-phase 1B also regulate degradation-related processes. Notably, Adr1p and Oaf1p regulate the expression of genes associated with peroxisomal functions (37,38); Rpn4p regulates proteasome genes (39); and Ume6p regulates autophagy (40).

Furthermore, a distinct group of TFs, including Gis1p, Rph1p, Adr1p, Cat8p, Gsm1p, Ert1p, Rds2p, Sip4p, Oaf1p, and Tog1p, orchestrates the metabolism of nonfermentable carbon sources such as glycerol, acetate, and fatty acids, facilitating

adaptation to glucose scarcity and optimizing energy utilization under these conditions (38,41,42); Ime1p, Ndt80p, and Ume6 were identified as regulators of initiation and progression for meiotic (43); and Gis1p activates the transcription of sporulation-specific genes (44).

Eigen-phase 2A

In eigen-phase 2A, the TFs were primarily associated with rRNA processing and ribosome biogenesis. In this eigen-phase, Dot6p, Tod6p, Stb3p, and Sfp1p are also the key regulators pivotal for maintaining and enhancing the efficiency of ribosome biogenesis (8-10). In addition, Reb1p and Mcm1p were identified as significant GRFs. Reb1p is essential for the transcriptional shift from rDNA to rRNA and regulates ribosome biogenesis genes (45,46), aligning with the observed enhancement of ribosome biogenesis in this eigen-phase [Figure 4B]. Mcm1p regulates genes critical for the cell cycle, cell wall structure, and arginine catabolism, highlighting its versatile impact on cellular functions and preparation for subsequent eigen-phases (47,48).

Eigen-phase 2B

In eigen-phase 2B, the TFs were mainly related to energy production pathways, including aerobic respiration and mitochondrial biogenesis. Central to this eigen-phase is the heme-activated protein complex Hap2p/3p/4p/5p, comprising Hap2p, Hap3p, and Hap5p, which plays a pivotal role in regulating the TCA cycle, the electron transport chain, ATP generation, and mitochondrial biogenesis (49). In addition, the mitochondrial protein Abf2p is crucial for maintaining mitochondrial DNA integrity, a fundamental aspect of mitochondrial health and function (50).

Furthermore, a distinct group of transcription factors, including Cat8, Rds2, and Sip4, are noted for their role in regulating the metabolism of nonfermentable carbon sources, highlighting the cell's adaptive mechanisms to efficient energy management under varying nutritional conditions (38).

The roles of key metabolites of each eigen-phase

Eigen-phase 1A

Inosine plays a crucial role in translation by modulating codon recognition and improving the efficiency and accuracy of protein synthesis (51). It is particularly important at the wobble position of tRNA anticodons, where inosine enables

noncanonical base pairings with multiple codons. Furthermore, inosine residues in mRNAs can directly alter the sequence of the translated polypeptide or influence the stability, localization, and splicing of transcripts.

Cystathionine is an intermediate in the methionine biosynthesis pathway and plays a crucial role in sulfur amino acid metabolism in yeast (4).

Inosinic acid, or inosine monophosphate (IMP), is a nucleotide that can be hydrolyzed to inosine. In addition, IMP is a crucial intermediate in the biosynthesis of purine nucleotides and further contributes to the synthesis of both adenine and guanine nucleotides.

Citrulline is a key intermediate in the urea cycle and a precursor in the biosynthesis of arginine, thereby playing a role in nitrogen metabolism in yeast.

Acetyl-CoA is a key intermediate in the TCA cycle, initiating a series of reactions that produce ATP, NADH, and FADH2, which are essential for cellular energy. The high biosynthetic activity in eigen-phase 1A demands substantial energy, which acetyl-CoA supports through its role in the TCA cycle. The ATP generated during this eigen-phase is crucial for powering the energy-intensive processes of translation and cell growth. In addition, the elevated transcriptional activity required for protein synthesis in eigen-phase 1A may be facilitated by acetyl-CoA through its involvement in histone acetylation. The availability of acetyl-CoA during eigen-phase 1A may also serve as a metabolic indicator, signaling sufficient energy and precursor molecules for biomolecule synthesis.

Eigen-phase 1B

Aspartate is a crucial component in the transport and generation of oxaloacetate for gluconeogenesis, playing a vital role in energy metabolism (52). Aspartate is transported from the mitochondria to the cytosol via the Agc2p, a process essential for maintaining the supply of oxaloacetate for gluconeogenesis.

Spermidine has been shown to promote longevity in various organisms by inhibiting oxidative stress, suppressing necrosis, and enhancing autophagy (53).

2−hydroxy−isovalerate, norvaline, aspartate, cytidine, and uridine are involved in the biosynthesis of various amino acids and nucleotides, preparing for the next eigen-phase.

Myo-inositol contributes to the osmotic stress response in yeast by acting as an osmolyte and participating in the synthesis of inositol-containing compounds that help maintain cellular osmoregulation (54).

Eigen-phase 2A

5-Aminoimidazole-4-carboxamide ribonucleotide (AICAR) is a key intermediate in purine biosynthesis and is essential for both DNA and RNA synthesis. In addition, AICAR stimulates the activity of AMP-dependent protein kinase (AMPK), which promotes glucose uptake and enhances glucose oxidation to produce energy (55). The role of AICAR in energy mechanisms may help coordinate the metabolic shifts in eigen-phase 2A, ensuring that energy production is optimized to meet cellular demands.

Similar to its role in eigen-phase 1A, the involvement of acetyl-CoA in energy production, biosynthesis, and gene regulation makes it a key player in the metabolic processes characteristic of eigen-phase 2A in the YMC.

Isocitrate plays a crucial role in yeast metabolism, primarily through its involvement in the tricarboxylic acid cycle (TCA cycle), which is central to cellular energy production and biosynthesis. In addition, the conversion of isocitrate to α-ketoglutarate provides an important precursor for the synthesis of glutamate, a key amino acid that serves as a nitrogen donor in the biosynthesis of other amino acids and nucleotides. The dual role of isocitrate in both energy production and biosynthesis exemplifies the metabolic flexibility required during eigen-phase 2A. Cells must balance ATP production with the generation of biosynthetic precursors, adjusting metabolic fluxes to optimize growth readiness.

Eigen-phase 2B

Methylcitrate, although less common than its relative metabolite citrate in yeast, functions in specific metabolic contexts, particularly in the processing of propionyl-CoA via the methylcitrate cycle. Methylcitrate is subsequently converted into pyruvate and succinate, both of which can enter the TCA cycle for energy production.

6-Phosphogluconate plays a significant role in the pentose phosphate pathway (PPP), which is crucial for maintaining cellular redox balance.

Aminolevulinic acid (ALA) is a key precursor in the biosynthesis of heme, a vital component of cytochromes and other hemoproteins involved in electron transport and various enzymatic reactions. The demand for heme increases during eigen-phase 2B due to intensified respiratory activity and the need for efficient electron transport. As a precursor to heme, ALA is crucial for ensuring adequate synthesis of hemoproteins necessary for these processes.

2-Methylmalate is an intermediate in the catabolism of branched-chain amino acids

(BCAAs) such as isoleucine and also functions in the methylcitrate cycle.

Cytidine monophosphate (CMP) and Guanosine monophosphate (GMP) are nucleotides that serve as essential building blocks of RNA, suggesting RNA breakdown during eigen-phase 2B.

Supplementary Figures

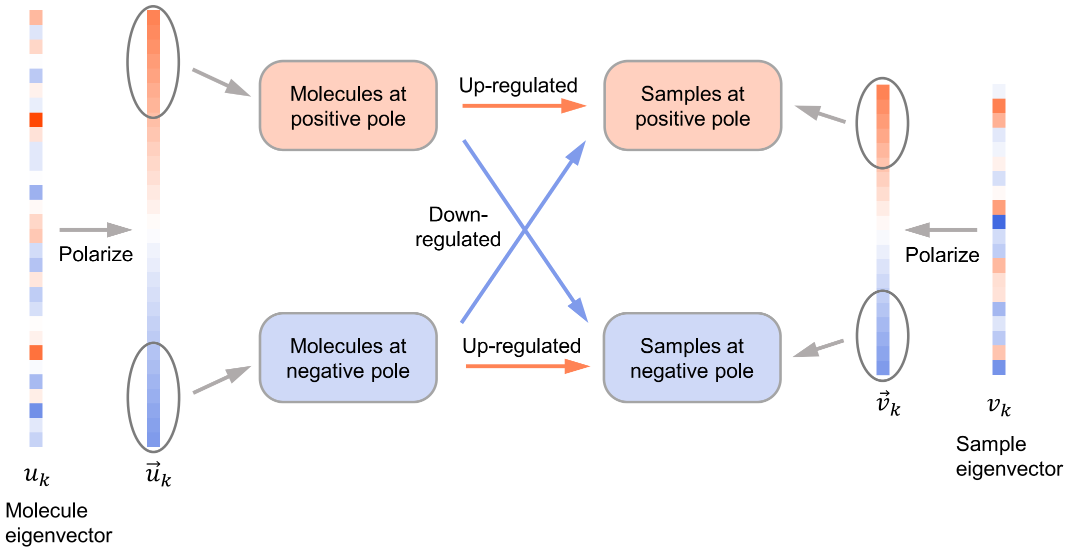

Figure S1. The schematic representation of dual eigen-analysis.

Molecule-eigenvector u_k and sample-eigenvector v_k are polarized into $\vec{u}_k$ and $\vec{v}_k$ by arranging their loadings in ascending order. Molecules at the positive pole of $\vec{u}_k$ exhibit up-regulation in samples at the positive pole of $\vec{v}_k$, and down-regulation in samples at the negative pole of $\vec{v}_k$. Conversely, the molecules at the negative pole of $\vec{u}_k$ exhibit down-regulation in samples at the positive pole of $\vec{v}_k$, and up-regulation in samples at the negative pole of $\vec{v}_k$.

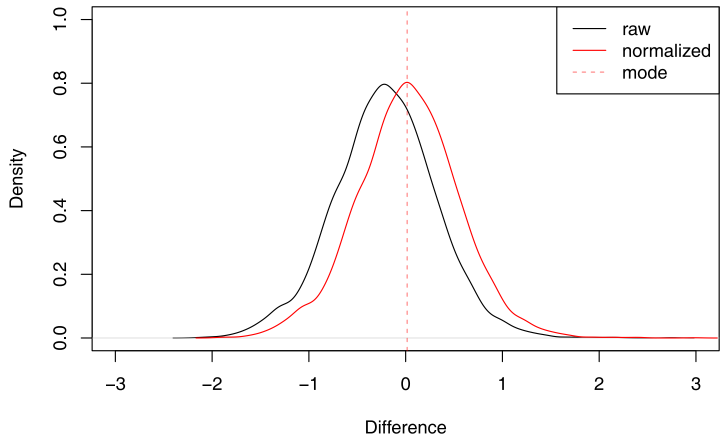

Figure S2. Densities of pairwise differences between samples T1 and T7 of epigenome H3K9ac before and after normalization by MUREN.

The mode of the density of pairwise differences is transformed to near zero after normalization, indicating the effectiveness of MUREN.

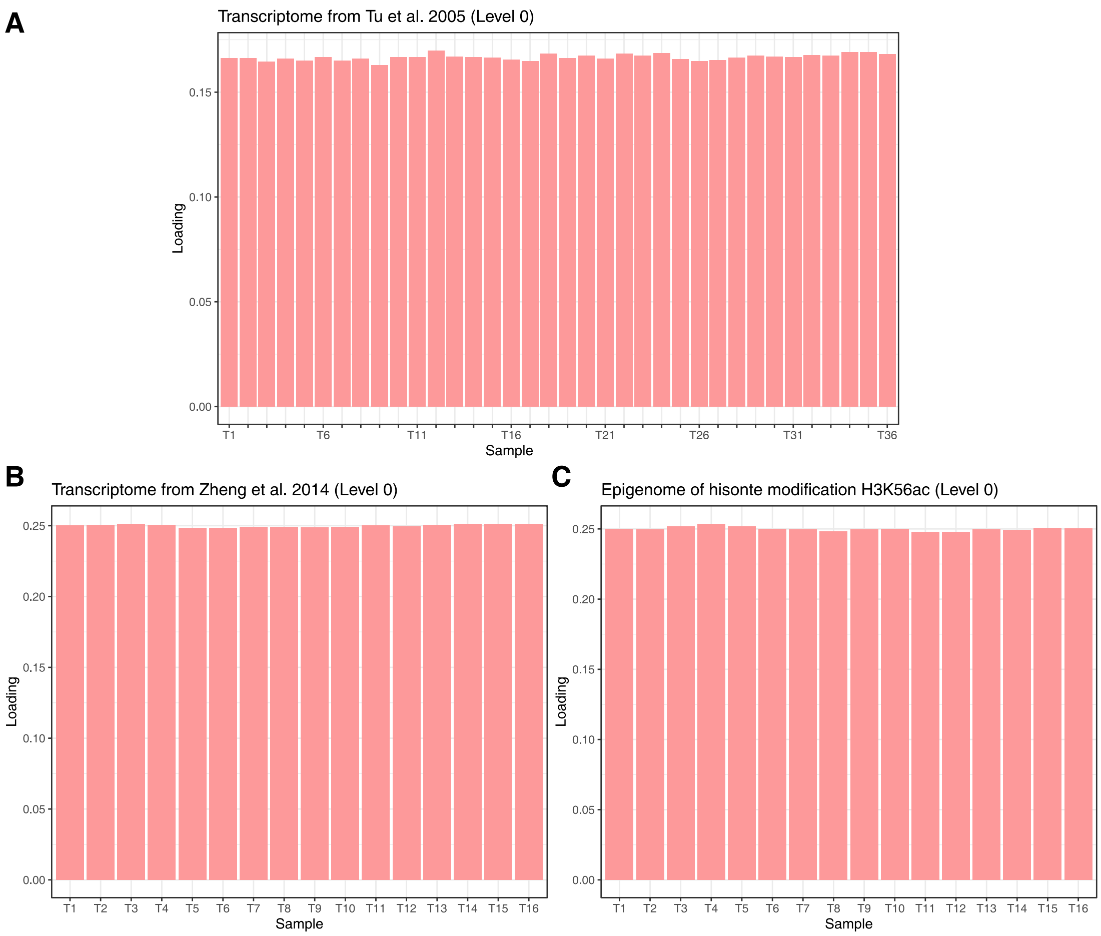

Figure S3. Loadings of the 0-th sample-eigenvector of omics data.

The sample loadings are nearly identical in omics datasets.

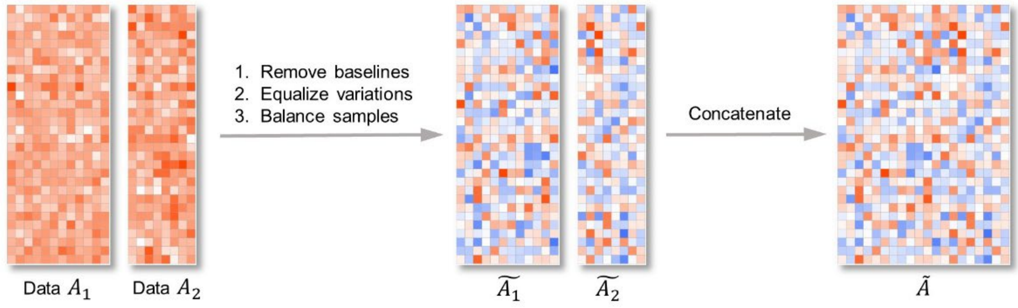

Figure S4. The preprocessing and concatenation of two expression profile matrices.

Two expression matrices with common genes are denoted as A_1 and A_2. Before concatenation, several preprocessing steps were undertaken. Firstly, their scales were aligned by removing baselines, specifically, by subtracting the 0-th eigen matrix as determined by SVD. Secondly, their variations were equalized by adjusting the F-norm, ensuring the densities of the two matrices approximated each other. Thirdly, to mitigate the impact of varying data sample sizes, each matrix was divided by the square root of its sample number. As a result, we obtained the preprocessed expression matrices $\widetilde{A_1}$ and $\widetilde{A_2}$. Next, these matrices were concatenated to form a single matrix $\tilde{A} = [\widetilde{A_1}\ \widetilde{A_2}]$.

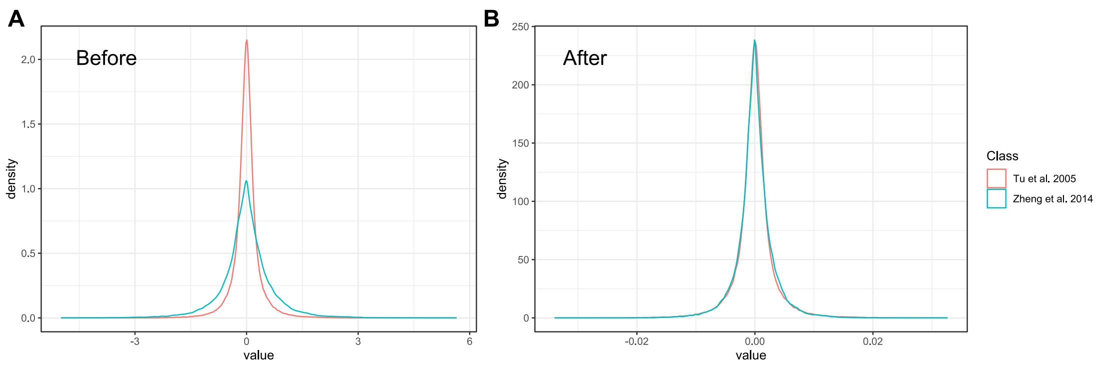

Figure S5. Densities of two transcriptomics datasets before and after scaled by F-norm.

The baselines of both transcriptomics datasets were removed. After scaling, the densities were almost identical.

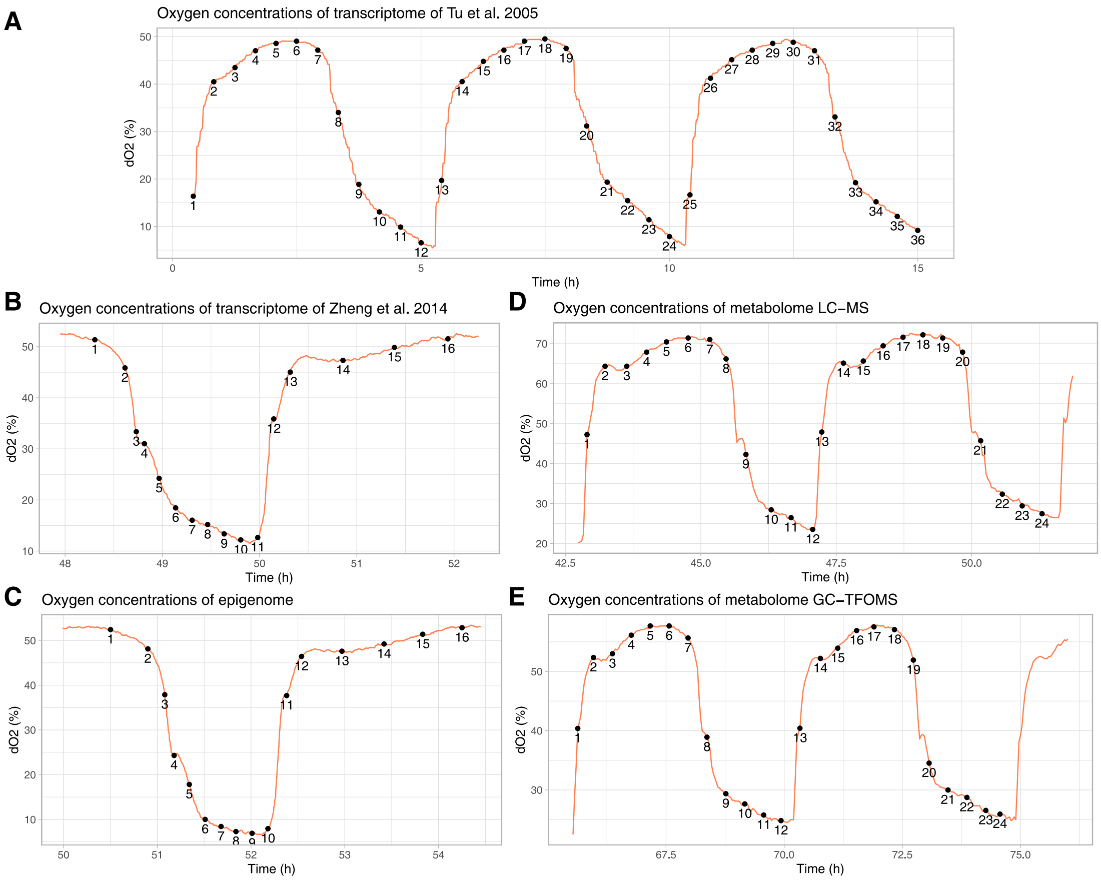

Figure S6. Oxygen concentration curves of each omics data.

(A) The concentration curve was obtained from Figure 2A in (2), and the sampling time points were obtained as it was sampled every 25 minutes. (B) The concentration curve was obtained from Figure 1A in (1), and the sampling time points were directly obtained from its Supplementary Table 1. (C) The concentration curve was obtained from Figure 3A in (1), and the sampling time points were directly obtained from its Supplementary Table 3. (D-E) The concentration curve and the sampling time points were directly obtained from Supplementary Table in (4).

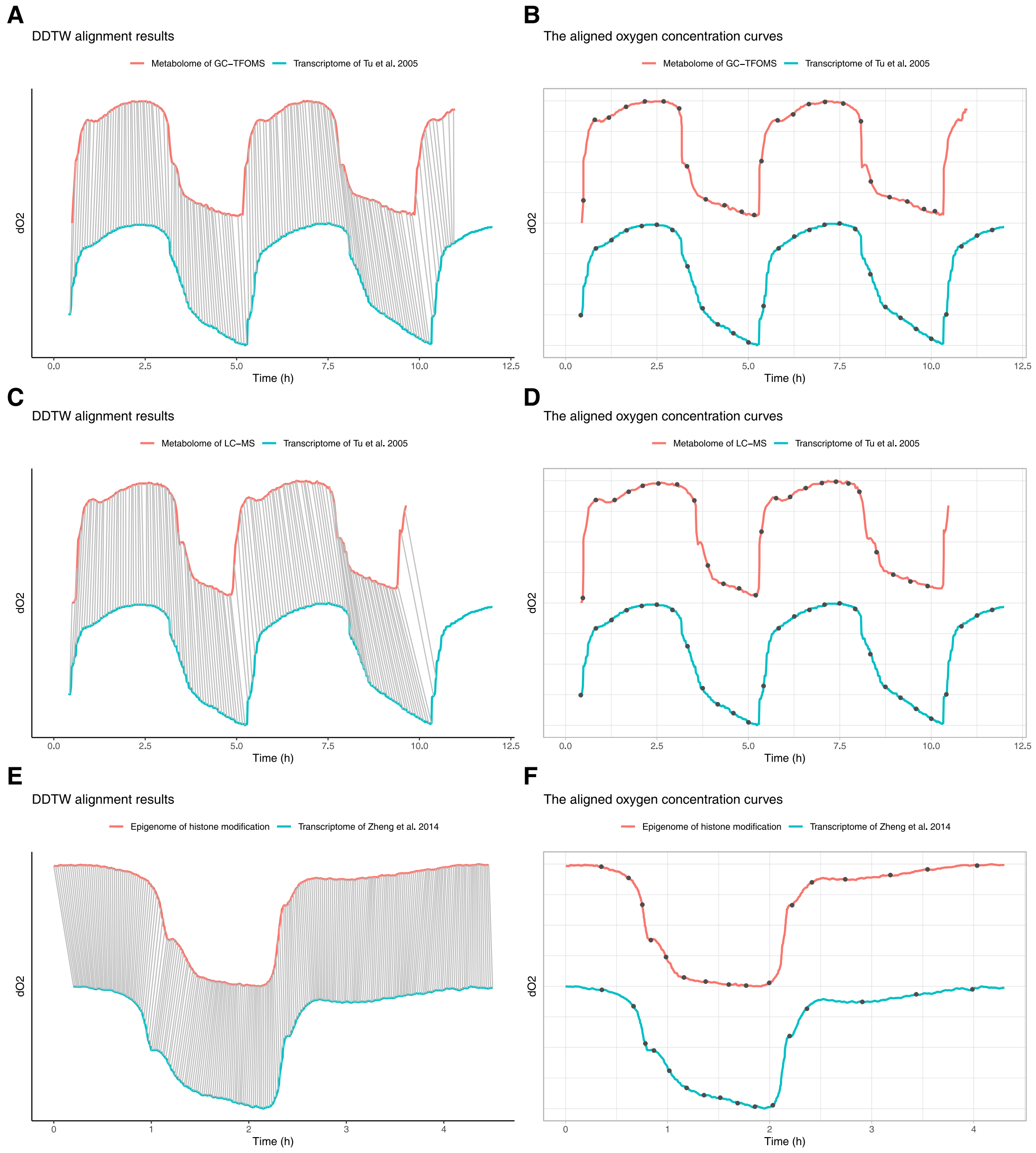

Figure S7. Oxygen concentration curves of different omics data after DDTW alignment.

The left plots show the derivative dynamic time warping (DDTW) mapping results of different oxygen concentration curves. The right plots show the aligned oxygen concentration curves and the black points are sampling time points.

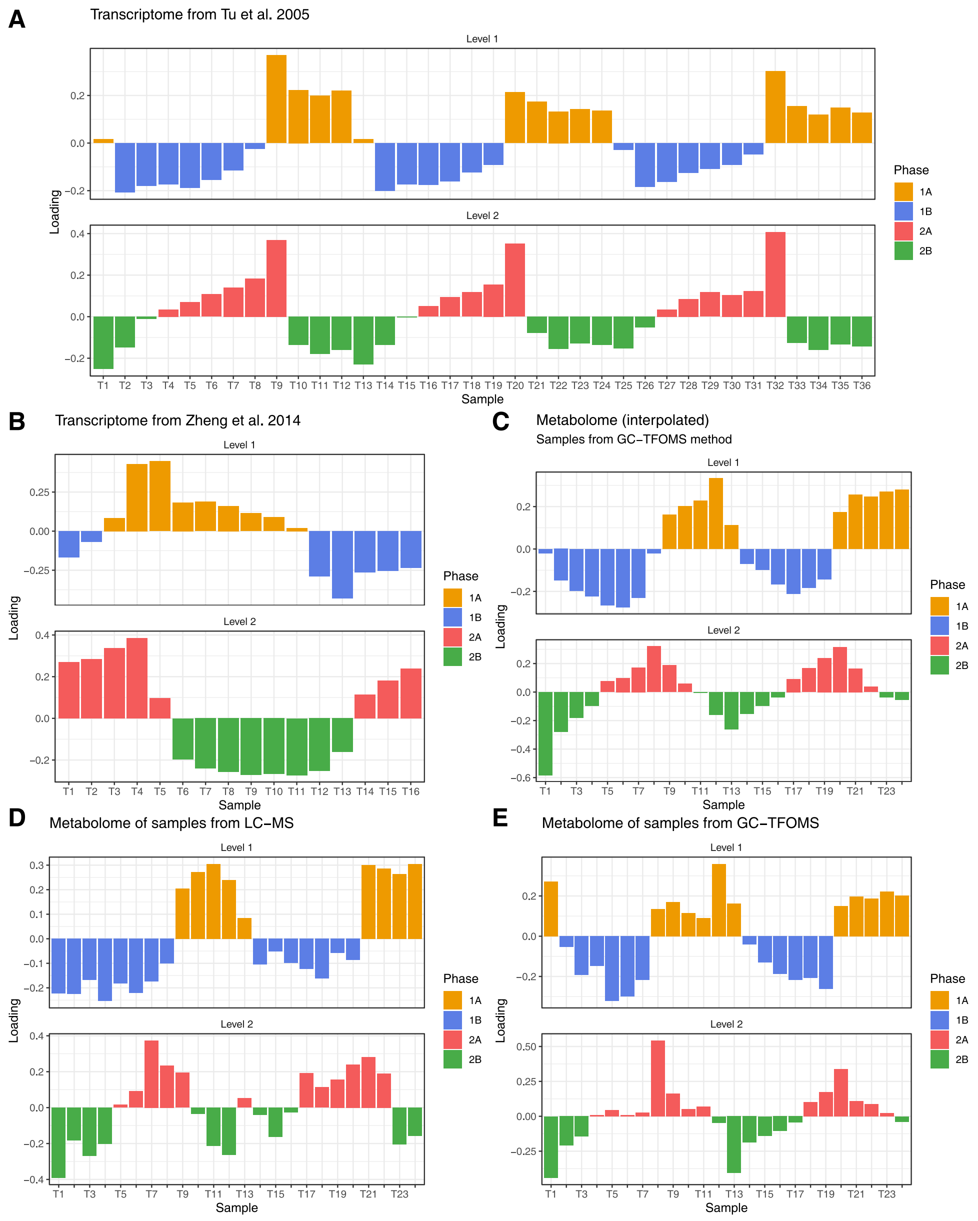

Figure S8. Loadings of the sample-eigenvectors at the top two levels in transcriptome and metabolome datasets.

The samples are arranged chronologically. These four eigen-phases are termed 1A, 1B, 2A, and 2B, corresponding to positive and negative sample loadings at levels 1 and 2. They are represented by orange, blue, red, and green, respectively.

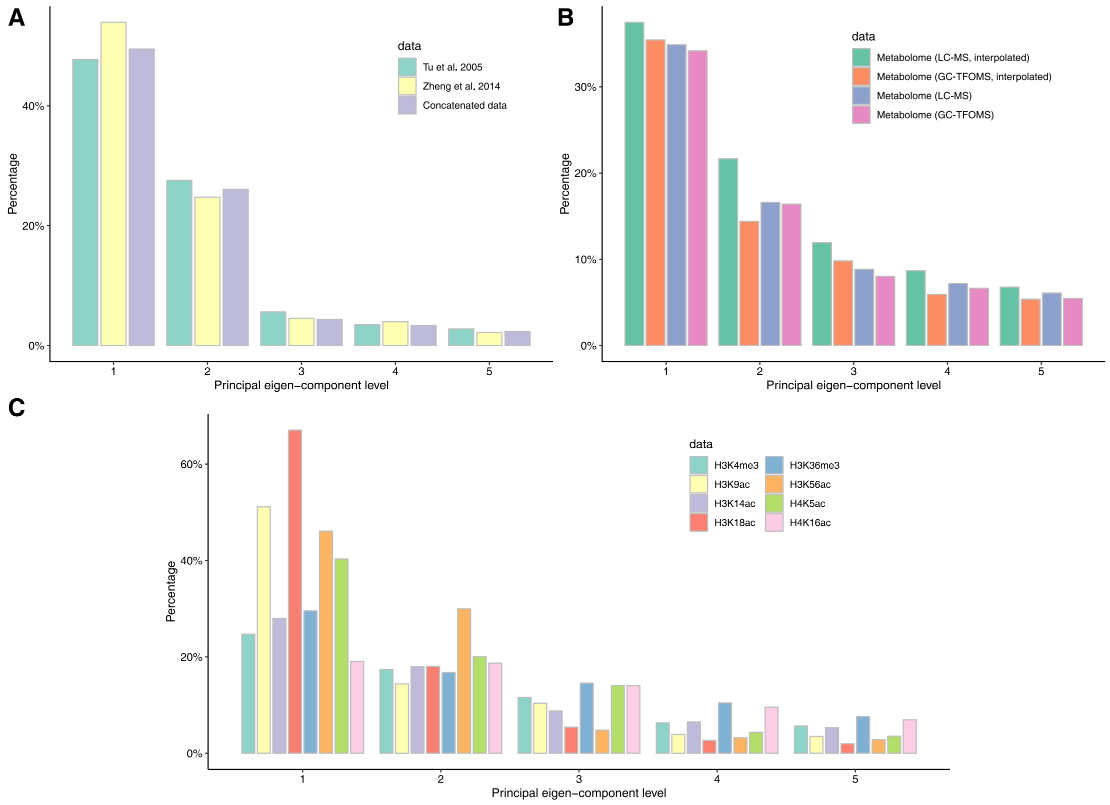

Figure S9. Percentages of squared singular values of the top five eigen-components of omics data.

The relative contributions of each level to the data were quantified by the ratio of squared singular values. In transcriptome, epigenome (for H3K9ac, H3K18ac, H3K56ac, and H4K5ac), and metabolome, the top two levels (1 and 2) contributed mainly with their cumulative ratios were over 50%.

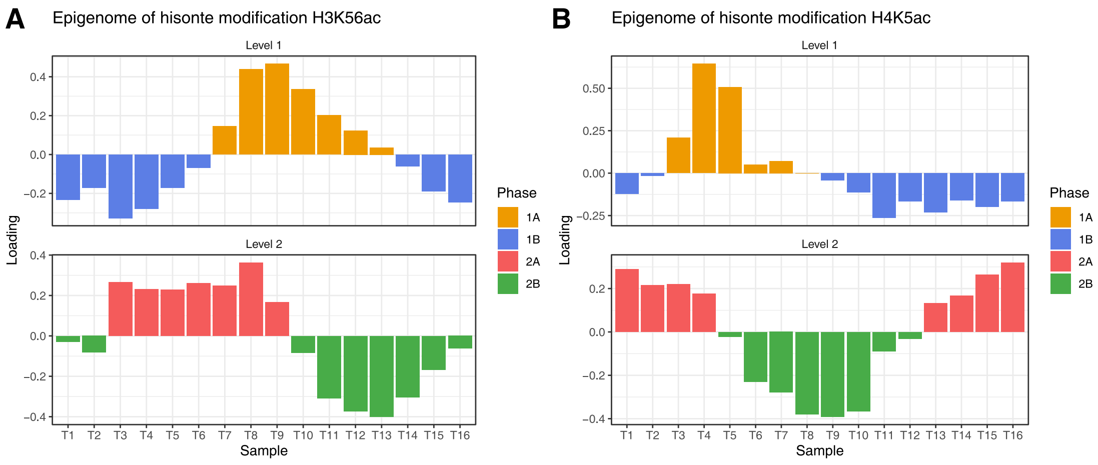

Figure S10. Loadings of the sample-eigenvectors at the top two levels in epigenome datasets for H3K56ac and H4K5ac.

The samples are arranged chronologically. These four eigen-phases are termed 1A, 1B, 2A, and 2B, corresponding to positive and negative sample loadings at levels 1 and 2. They are represented by orange, blue, red, and green, respectively.

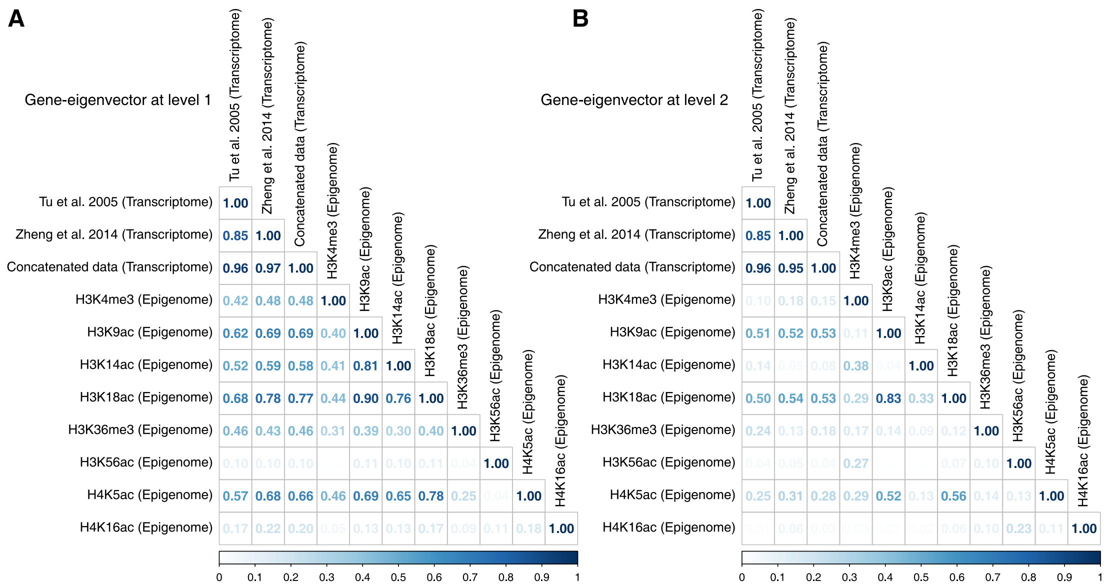

Figure S11. Pairwise Pearson correlation coefficients between gene-eigenvectors of transcriptome and epigenome at the top two levels.

The correlation coefficients of gene-eigenvectors between transcriptomes were high (>0.85). The gene-eigenvectors of the epigenome for H3K9ac and H3K18ac exhibited higher correlation coefficients (>0.5) with that of transcriptome.

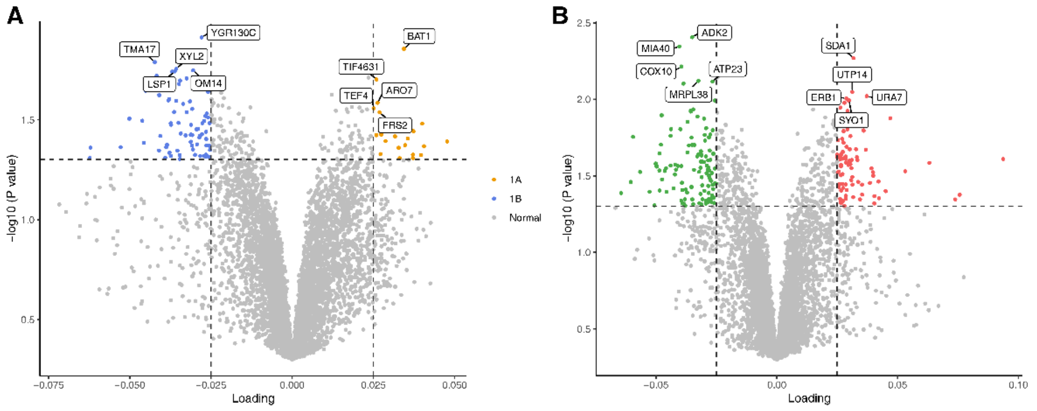

Figure S12. Volcano plots of the marker genes in each eigen-phase.

The threshold for p-values is 0.05 and for loadings is 0.025 (or -0.025)

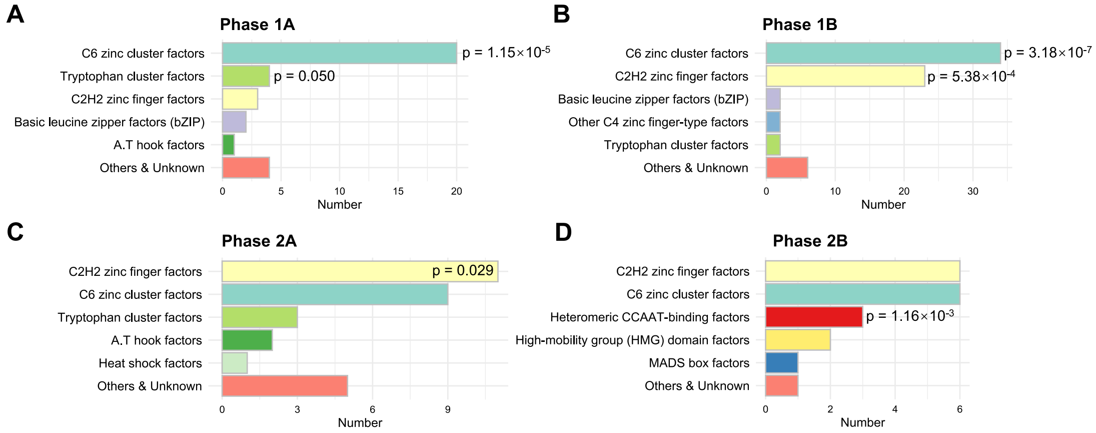

Figure S13. Counts for classifications of significant TFs in each eigen-phase.

The significance classes in each eigen-phase were determined by contingency table tests. The p-values less than 0.05 were shown at the corresponding class in the plot.

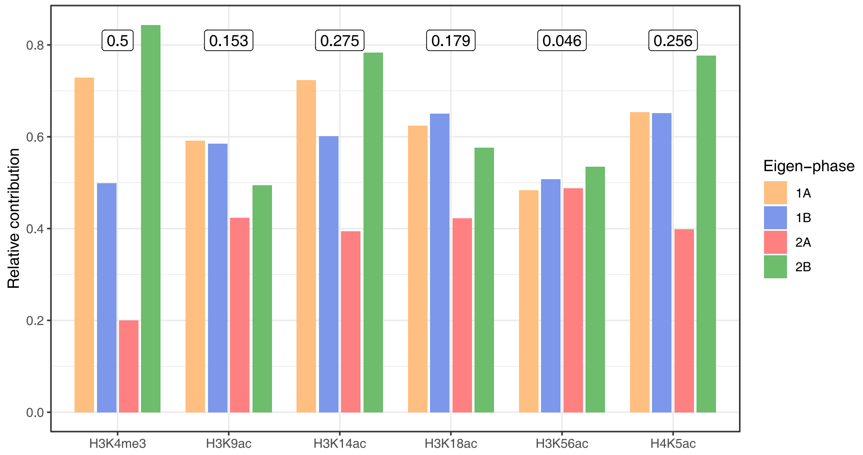

Figure S14. Relative contribution of histone modifications to each eigen-phase

The numbers in the upper box represent the coefficient of variance (CV). The smaller the CV, the more uniform the contribution to each eigen-phases.

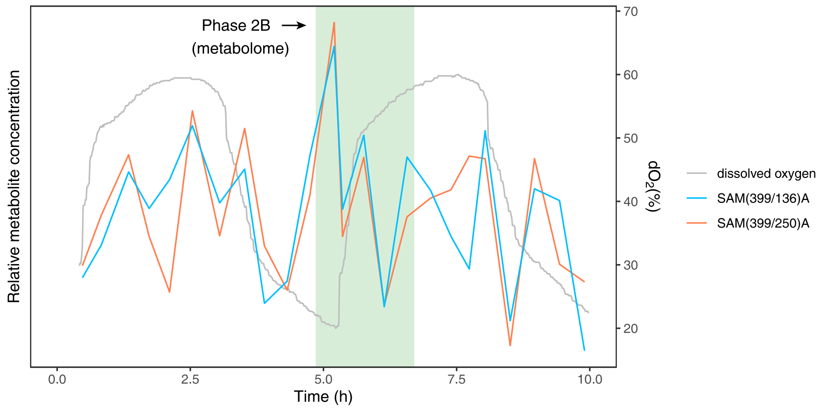

Figure S15. Relative concentration of S-adenosylmethionine (SAM).

Its concentration peaks at the eigen-phase 2B (green background).

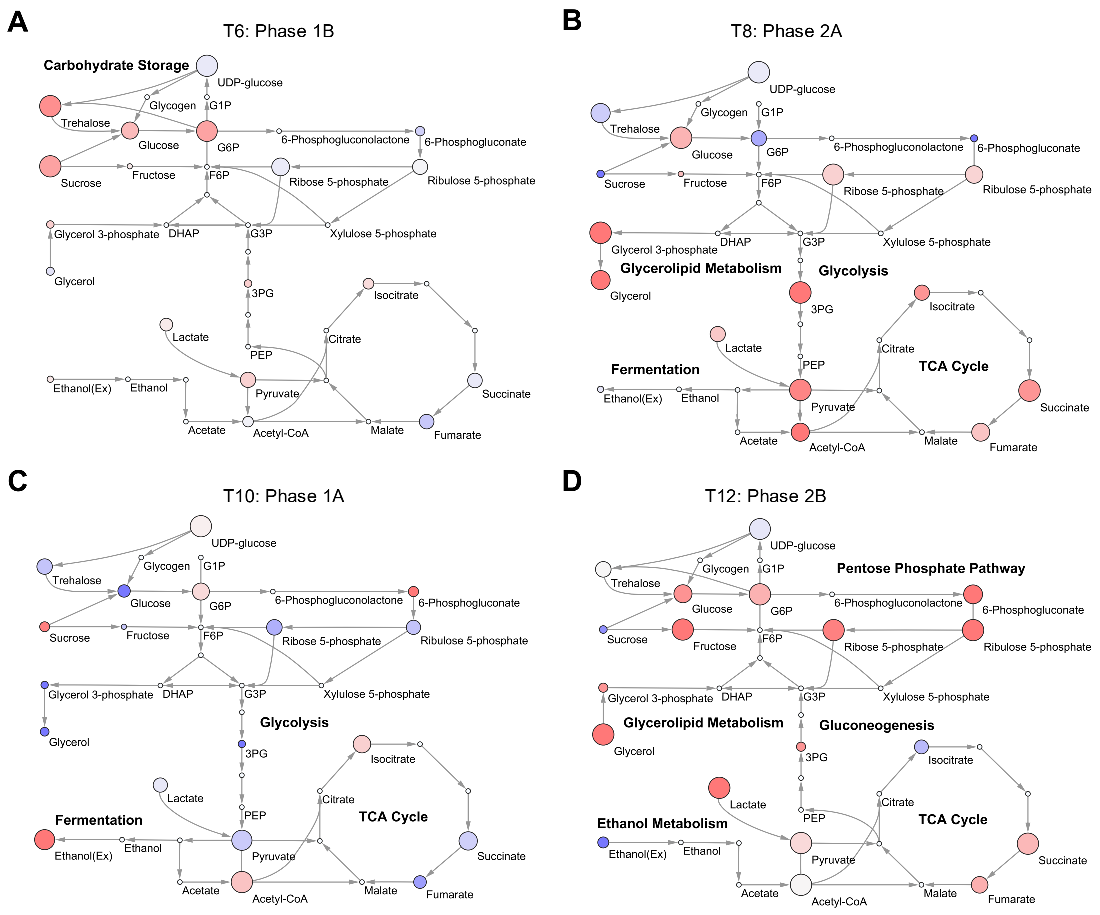

Figure S16. Central carbon metabolism network of four eigen-phases.

The size of the metabolite circle represents their current amount. The larger the size, the greater the amount. The color represents the difference in amount relative to the previous eigen-phase. Specifically, the difference of T6 to T3, T8 to T6, T10 to T8, and T12 to T10, respectively. Red represents an increase, and blue represents a decrease. The deeper the color, the greater the change. G1P, Glucose 1-phosphate; G6P, Glucose 6-phosphate; F6P, Fructose 6-phosphate; DHAP, Dihydroxyacetone phosphate; G3P, Glycerol-3-phosphate; 3PG, 3-Phosphoglyceric acid; PEP, Phosphoenolpyruvate; Ex, extracellular.

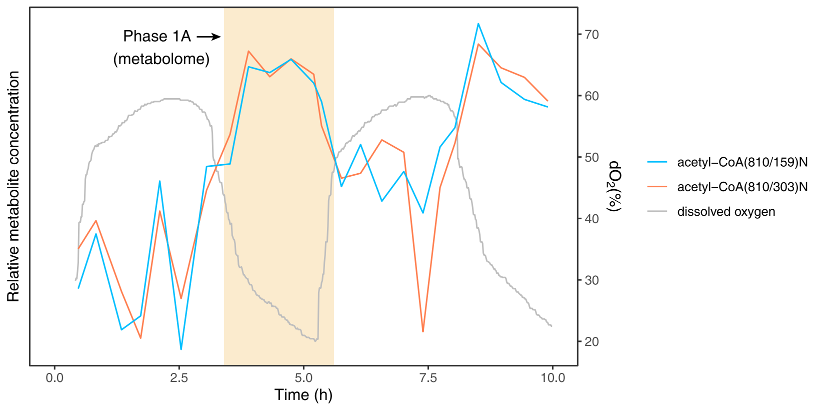

Figure S17. Relative concentration of acetyl-CoA.

Its concentration is at a high level during the metabolomic eigen-phase 1A (orange).

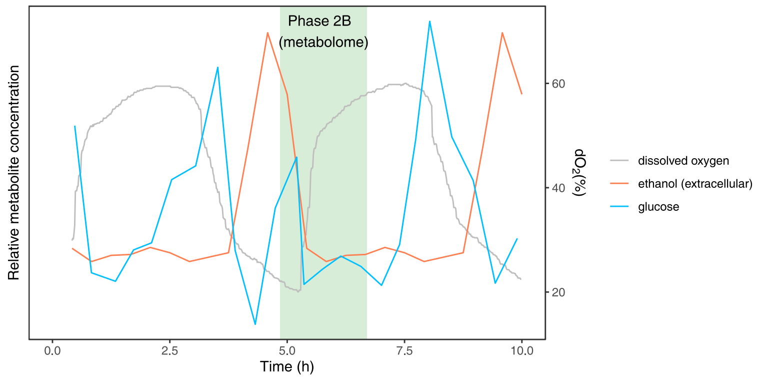

Figure S18. Relative concentration of ethanol (extracellular) and glucose.

Following the reduction of glucose, the concentration of extracellular ethanol peaks at the beginning of eigen-phase 2B (green background), and soon it drops, indicating the reabsorption and consumption of ethanol.

Supplementary Tables

Table S1. Summary of YMC omics datasets.

Data type	Method	Time points	Cycles	Publications	Data resource
Transcriptome	Microarray	36	3	(2)	GSE3431
Transcriptome	RNA-seq	16	1	(1)	GSE52339
Epigenome	ChIP-seq	16	1	(1,56)	GSE52339; GSE118889
Metabolome	LC-MS & GC-TOFMS	24	2	(4)	Supplementary

Table S2. Maximum cross-correlation of sample-eigenvectors between transcriptome and other omics datasets at the top two level.

Dataset	Level	Maximum cross-correlation
Metabolome	1	0.919
	2	0.672
H3K4me3	1	0.789
	2	0.649
H3K9ac	1	0.960
	2	0.898
H3K14ac	1	0.937
	2	0.458
H3K18ac	1	0.977
	2	0.967
H3K36me3	1	0.569

	2	0.466
H3K56ac	1	0.743
	2	0.782
H4K5ac	1	0.894
	2	0.925
H4K16ac	1	0.587
	2	0.748

Table S3. Time differences between different omics datasets.

From	To	Level	Time difference (minute)	95% Confidence interval
Epigenome for H3K9ac	Transcriptome (concatenated)	1	3.66	[2.76, 4.68]
		2	3.48	[1.98, 5.10]
Epigenome for H3K18ac	Transcriptome (concatenated)	1	3.18	[2.40, 4.02]
		2	3.48	[1.92, 5.04]
Transcriptome (concatenated)	Metabolome (Interpolated to LC-MS)	1	11.64	[9.24, 14.16]
		2	13.80	[4.50, 27.48]
Metabolome (Interpolated to LC-MS)	Transcriptome, next eigen-phase (concatenated)	1	32.88	[29.64, 36.30]
		2	43.50	[36.30, 53.70]

Table S4. Marker genes of eigen-phase 1A.

Gene	GeneTitle	Loading	P-value
BAT1	Branched-chain Amino acid Transaminase	0.0344	0.0140
TIF4631	Translation Initiation Factor	0.0260	0.0199
ARO7	AROmatic amino acid requiring	0.0263	0.0260
TEF4	Translation Elongation Factor	0.0251	0.0277
FRS2	phenylalanyl (F)-tRNA Synthetase	0.0269	0.0291
CTP1	Citrate Transport Protein	0.0400	0.0331

CYS3	CYStathionine gamma-lyase	0.0354	0.0333
ELO3	fatty acid ELOngation	0.0329	0.0351
RPS22A	Ribosomal Protein of the Small subunit	0.0272	0.0352
IMD3	IMP Dehydrogenase	0.0297	0.0360
SHM2	Serine HydroxyMethyltransferase	0.0373	0.0360
FUR1	5-FluoroURidine resistant	0.0274	0.0374
VRG4	Vandate Resistance Glycosylation	0.0260	0.0377
SER33	SERine requiring	0.0259	0.0377
MIS1	MItochondrial C1-tetrahydrofolate Synthase	0.0317	0.0382
NOP56	NucleOlar Protein of 56.8 kDa	0.0356	0.0392
LEU9	LEUcine biosynthesis	0.0287	0.0404
SAM1	S-AdenosylMethionine requiring	0.0477	0.0405
LYS1	LYSine requiring	0.0348	0.0424
LYS4	LYSine requiring	0.0406	0.0428
LYS12	LYSine requiring	0.0328	0.0436
ROK1	Rescuer Of Kem1	0.0272	0.0469
RPS26B	Ribosomal Protein of the Small subunit	0.0372	0.0473
FSH1	Family of Serine Hydrolases	0.0397	0.0475
ADE17	ADEnine	0.0371	0.0491
GAR1	Glycine Arginine Rich	0.0331	0.0492

Table S5. Marker genes of eigen-phase 1B.

Gene	GeneTitle	Loading	P-value
YGR130C		-0.0279	0.0122
TMA17	Translation Machinery Associated	-0.0423	0.0163
XYL2		-0.0357	0.0176
OM14	Outer Membrane Protein of 14 kDa	-0.0305	0.0179
LSP1	Long chain bases Stimulate Phosphorylation	-0.0362	0.0181
MHO1	Memo HOmolog	-0.0369	0.0182
DCS2	DeCapping Scavenger	-0.0417	0.0191
ATH1	Acid TreHalase	-0.0325	0.0196
SDS24	homolog of S. pombe SDS23	-0.0345	0.0201
YDR018C		-0.0271	0.0209
GUT2	Glycerol UTilization	-0.0349	0.0209
TSL1	Trehalose Synthase Long chain	-0.0451	0.0226
SGA1	Sporulation-specific GlycoAmylase	-0.0259	0.0229
YIR016W		-0.0409	0.0238

YGR127W		-0.0379	0.0250
GPX1	Glutathione PeroXidase	-0.0382	0.0253
GID11	Glucose Induced Degradation deficient	-0.0357	0.0254
EIS1	EISosome	-0.0315	0.0256
TPS1	Trehalose-6-Phosphate Synthase	-0.0336	0.0263
SAF1	SCF Associated Factor	-0.0308	0.0279
XBP1	XhoI site-Binding Protein	-0.0364	0.0289
IGD1	Inhibitor of Glycogen Debranching	-0.0324	0.0290
PRC1	PRoteinase C	-0.0263	0.0294
VLD1	Vacuole Localized Dsc protein	-0.0267	0.0303
AIM19	Altered Inheritance rate of Mitochondria	-0.0259	0.0308
GIP2	Glc7-Interacting Protein	-0.0501	0.0312
PHM7	PHosphate Metabolism	-0.0462	0.0320
YMR262W		-0.0271	0.0321
PGM2	PhosphoGlucoMutase	-0.0350	0.0328
TPK2	Takashi's Protein Kinase	-0.0254	0.0329
TPS2	Trehalose-6-Phosphate Synthase/phosphatase	-0.0374	0.0330
YBR085C-A		-0.0277	0.0330
STF2	STabilizing Factor	-0.0373	0.0335
PIL1	Phosphorylation Inhibited by Long chain bases	-0.0311	0.0335
ATG39	AuTophaGy related	-0.0290	0.0351
PFK26	6-PhosphoFructo-2-Kinase	-0.0295	0.0353
GLG1	Glycogenin-Like Gene	-0.0265	0.0363
SDD1	Suppressor of Degenerative Death	-0.0343	0.0370
ARA1	D-ARAbinose dehydrogenase	-0.0278	0.0373
GDB1	Glycogen DeBranching	-0.0312	0.0375
PNC1	Pyrazinamidase and NiCotinamidase	-0.0308	0.0377
YKL091C		-0.0328	0.0378
GRE3	Genes de Respuesta a Estres (stress responsive genes)	-0.0284	0.0380
GSY2	Glycogen SYnthase	-0.0406	0.0381
OPI10	OverProducer of Inositol	-0.0286	0.0388
ATG8	AuTophaGy related	-0.0265	0.0394
YJL163C		-0.0312	0.0407
SPS19	SPorulation-Specific	-0.0457	0.0412
ECL1	Extends Chronological Lifespan	-0.0293	0.0420
OM45	Outer Membrane	-0.0385	0.0422
SDP1	Stress-inducible Dual specificity Phosphatase	-0.0264	0.0425
NNR2	Nicotinamide Nucleotide Repair	-0.0309	0.0425

MRX18	Mitochondrial oRganization of gene eXpression (MIOREX)	-0.0256	0.0426
NDE2	NADH Dehydrogenase, External	-0.0528	0.0433
GPH1	Glycogen PHosphorylase	-0.0388	0.0434
RGI2	Respiratory growth induced	-0.0621	0.0436
TPK1	Takashi's Protein Kinase	-0.0306	0.0445
UBP15	UBiquitin-specific Protease	-0.0253	0.0447
JEN1		-0.0410	0.0451
NCE102	NonClassical Export	-0.0354	0.0451
TFS1	cdc Twenty-Five Suppressor	-0.0378	0.0462
GGA1	Golgi-localized, Gamma-adaptin ear homology, Arf-binding protein	-0.0265	0.0473
GLK1	GLucoKinase	-0.0299	0.0473
MDH2	Malate DeHydrogenase	-0.0346	0.0474
YMR196W		-0.0374	0.0479
ATG7	AuTophaGy related	-0.0271	0.0479
UBX6	UBiquitin regulatory X	-0.0283	0.0480
RNY1	RiboNuclease from Yeast	-0.0266	0.0483
GSP2	Genetic Suppressor of Prp20-1	-0.0257	0.0484
FAA1	Fatty Acid Activation	-0.0391	0.0490
FAT3	FATty acid transporter 3	-0.0623	0.0491
YPL113C		-0.0346	0.0496

Table S6. Marker genes of eigen-phase 2A.

Gene	GeneTitle	Loading	P-value
SDA1	Severe Depolymerization of Actin	0.0317	0.0054
UTP14	U Three Protein	0.0312	0.0090
URA7	URAcil requiring	0.0372	0.0095
ERB1	Eukaryotic Ribosome Biogenesis	0.0288	0.0099
SYO1	SYnchronized impOrt or SYmpOrtin	0.0299	0.0101
CNS1	CyclophiliN Seven suppressor	0.0297	0.0102
MRD1	Multiple RNA-binding domain	0.0280	0.0106
ECM16	ExtraCellular Mutant	0.0264	0.0113
MAK5	MAintenance of Killer	0.0293	0.0119
SHC1	Sporulation-specific Homolog of CSD4	0.0470	0.0133
BMT5	Base Methyltransferase of Twenty five S rRNA 5	0.0318	0.0137
UTP25	U Three Protein	0.0264	0.0138
FAF1	Forty (40) S Assembly Factor	0.0252	0.0140

RNT1	RNase Three	0.0313	0.0145
TSR1	Twenty S rRNA accumulation	0.0292	0.0157
ECM1	ExtraCellular Mutant	0.0359	0.0160
CGR1	Coiled-coil Growth-Regulated	0.0360	0.0160
RIX1	RIbosome eXport	0.0276	0.0161
ARX1	Associated with Ribosomal eXport complex	0.0305	0.0174
BAS1	BASal	0.0270	0.0181
BMS1	BMh Sensitive	0.0253	0.0185
TPO1	Transporter of POlyamines	0.0276	0.0192
		0.0288	0.0204
HCA4	Helicase CA	0.0328	0.0212
DRS1	Deficiency of Ribosomal Subunits	0.0302	0.0212
PRP43	Pre-mRNA Processing	0.0369	0.0213
LTV1	Low Temperature Viability	0.0273	0.0220
GCD10	General Control Derepressed	0.0267	0.0227
RPA43	RNA Polymerase A	0.0267	0.0229
RPF1	Ribosome Production Factor	0.0251	0.0230
RIX7	RIbosome eXport	0.0260	0.0233
NOP8	NucleOlar Protein	0.0265	0.0234
FAL1	eukaryotic translation initiation factor Four A Like	0.0284	0.0236
NDE2	NADH Dehydrogenase, External	0.0332	0.0238
NIP7	Nuclear ImPort	0.0251	0.0239
NMD3	Nonsense-Mediated mRNA Decay	0.0285	0.0243
SAS10	Something About Silencing	0.0272	0.0244
GRE1	Genes de Respuesta a Estres (spanish for stress responsive genes)	0.0937	0.0246
GTT1	GlutaThione Transferase	0.0263	0.0246
DHR2	DEAH-box RNA helicase	0.0334	0.0249
RPF2	Ribosome Production Factor	0.0310	0.0249
ENP2	Essential Nuclear Protein	0.0301	0.0251
RPA49	RNA Polymerase A	0.0287	0.0259
PMA2	Plasma Membrane ATPase	0.0632	0.0260
YVH1	Yeast vaccinia virus VH1 Homolog	0.0305	0.0265
TRM11	TRna Methyltransferase	0.0297	0.0265
RFU1	Regulator of Free Ubiquitin chains	0.0296	0.0268
RRB1	Regulator of Ribosome Biogenesis	0.0266	0.0268
PUF6	PUmilio-homology domain Family	0.0266	0.0274
YTM1		0.0309	0.0281
GFD2	Great for Full DEAD box protein activity	0.0392	0.0282
		0.0532	0.0295

NOP7	NucleOlar Protein	0.0256	0.0296
FAT3	FATty acid transporter 3	0.0399	0.0299
FUI1	5-FlUorourIdine resistance	0.0252	0.0302
ARB1	ATP-binding cassette protein involved in Ribosome Biogenesis	0.0272	0.0308
IFH1	Interacts with Fork Head	0.0265	0.0314
DBP2	Dead Box Protein	0.0399	0.0315
UTP5	U Three Protein	0.0313	0.0322
RMT2	aRginine MeThyltransferase	0.0308	0.0338
DBP2	Dead Box Protein	0.0421	0.0344
RSA4	RiboSome Assembly	0.0337	0.0346
BUD20	BUD site selection	0.0386	0.0351
RPH1	Regulator of PHR1	0.0270	0.0357
UIP4	Ulp1 Interacting Protein	0.0258	0.0359
DBP3	Dead Box Protein	0.0286	0.0359
ERR3	Enolase-Related Repeat	0.0353	0.0361
ENP1	Essential Nuclear Protein	0.0313	0.0361
ROX3	Regulation by OXygen	0.0257	0.0374
RRT14	Regulator of rDNA Transcription	0.0275	0.0379
PHO90	PHOsphate metabolism	0.0289	0.0391
SPB1	Suppressor of PaB1 mutant	0.0266	0.0394
NQM1	Non-Quiescent Mutant	0.0451	0.0398
SSF1	Suppressor of ste4 (Four)	0.0353	0.0419
		0.0757	0.0420
RRP12	Ribosomal RNA Processing	0.0286	0.0427
GPP2	Glycerol-3-Phosphate Phosphatase	0.0404	0.0429
NOG1	NucleOlar G-protein	0.0316	0.0436
PIR3	Protein containing Internal Repeats	0.0423	0.0443
NOP14	NucleOlar Protein	0.0262	0.0447
MAK16	MAintenance of Killer	0.0286	0.0447
SSU1	Sensitive to SUlfite	0.0344	0.0451
BMT6	Base Methyltransferase of Twenty five S rRNA 6	0.0263	0.0451
HEF3	Homolog of EF-3	0.0739	0.0451
RRP36	Ribosomal RNA Processing	0.0273	0.0462
PNS1	pH Nine Sensitive	0.0265	0.0475
SPG4	Stationary Phase Gene	0.0404	0.0479
BFR2	BreFeldin A Resistance	0.0280	0.0496

Table S7. Marker genes of eigen-phase 2B.

Gene	GeneTitle	Loading	P-value
ADK2	ADenylate Kinase	-0.0350	0.0039
MIA40	Mitochondrial intermembrane space Import and Assembly	-0.0404	0.0045
COX10	Cytochrome c OXidase	-0.0395	0.0061
MRPL38	Mitochondrial Ribosomal Protein, Large subunit	-0.0325	0.0076
ATP23		-0.0266	0.0077
HXK1	HeXoKinase	-0.0386	0.0079
MRPL35	Mitochondrial Ribosomal Protein, Large subunit	-0.0358	0.0093
MRX14	Mitochondrial oRganization of gene eXpression (MIOREX)	-0.0436	0.0094
MSC6	Meiotic Sister-Chromatid recombination	-0.0329	0.0097
RSM24	Ribosomal Small subunit of Mitochondria	-0.0403	0.0098
NAM9	Nuclear Accommodation of Mitochondria	-0.0366	0.0100
MRPL19	Mitochondrial Ribosomal Protein, Large subunit	-0.0339	0.0101
MRP20	Mitochondrial Ribosomal Protein	-0.0256	0.0102
SCO1	Suppressor of Cytochrome Oxidase deficiency	-0.0346	0.0116
QRI5	Quasi-Renownless Information	-0.0356	0.0118
MZM1	Mitochondrial Zinc Maintenance	-0.0356	0.0120
TUF1		-0.0477	0.0127
MRPS12	Mitochondrial Ribosomal Protein, Small subunit	-0.0328	0.0130
ECM19	ExtraCellular Mutant	-0.0301	0.0134
MRF1	Mitochondrial peptide chain Release Factor	-0.0370	0.0140
ACO1	ACOnitase	-0.0292	0.0154
MRPL49	Mitochondrial Ribosomal Protein, Large subunit	-0.0438	0.0156
MRPL40	Mitochondrial Ribosomal Protein, Large subunit	-0.0399	0.0157
DIA4	Digs Into Agar	-0.0266	0.0159
COX11	Cytochrome c OXidase	-0.0276	0.0170
MDH1	Malate DeHydrogenase	-0.0298	0.0171
CYT2	CYTochrome-c1-heme-lyase	-0.0439	0.0173
TOS6		-0.0595	0.0176
MRPS16	Mitochondrial Ribosomal Protein, Small subunit	-0.0281	0.0177
NDI1	NADH Dehydrogenase Internal	-0.0353	0.0177
CBP3	Cytochrome B mRNA Processing	-0.0297	0.0186

IDH1	Isocitrate DeHydrogenase	-0.0479	0.0197
MSE1	Mitochondrial aminoacyl-tRNA Synthetase, Glutamate (E)	-0.0351	0.0198
MST1	Mitochondrial aminoacyl-tRNA Synthetase, Threonine	-0.0342	0.0199
COX14	Cytochrome c OXidase	-0.0364	0.0200
MRPL16	Mitochondrial Ribosomal Protein, Large subunit	-0.0278	0.0216
SDH1	Succinate DeHydrogenase	-0.0314	0.0218
RRF1	Ribosome Recycling Factor	-0.0370	0.0222
MRPL4	Mitochondrial Ribosomal Protein, Large subunit	-0.0486	0.0225
CBP4	Cytochrome B mRNA Processing	-0.0263	0.0226
SDH4	Succinate DeHydrogenase	-0.0325	0.0228
COQ1	COenzyme Q	-0.0311	0.0231
RTC6	Restriction of Telomere Capping	-0.0312	0.0232
ATP12	ATP synthase	-0.0293	0.0235
MSY1	Mitochondrial aminoacyl-tRNA Synthetase, tyrosine (Y)	-0.0329	0.0236
YML6		-0.0275	0.0248
SDH7	Succinate DeHydrogenase	-0.0284	0.0250
TOS4	Target Of Sbf	-0.0308	0.0255
QCR2	QH2:cytochrome-C oxidoReductase	-0.0314	0.0255
HXT16	HeXose Transporter	-0.0302	0.0257
SVS1	Suppressor of Vanadate Sensitivity	-0.0501	0.0263
MRPL32	Mitochondrial Ribosomal Protein, Large subunit	-0.0420	0.0268
MRPL7	Mitochondrial Ribosomal Protein, Large subunit	-0.0439	0.0271
MRPL22	Mitochondrial Ribosomal Protein, Large subunit	-0.0290	0.0271
MHR1	Mitochondrial Homologous Recombination	-0.0409	0.0272
RML2	Ribosomal Mitochondrial Large	-0.0395	0.0273
PET117	PETite colonies	-0.0499	0.0273
RNR1	RiboNucleotide Reductase	-0.0496	0.0275
POL1	POLymerase	-0.0283	0.0279
AIM10	Altered Inheritance rate of Mitochondria	-0.0316	0.0280
CLN1	CycLiN	-0.0459	0.0282
COA1	Cytochrome Oxidase Assembly	-0.0256	0.0283
OMS1	OXA1 Multicopy Suppressor	-0.0417	0.0288
HSP10	Heat Shock Protein	-0.0285	0.0288
MRP17	Mitochondrial Ribosomal Protein	-0.0343	0.0289

CDC21	Cell Division Cycle	-0.0280	0.0291
MEF1	Mitochondrial Elongation Factor	-0.0486	0.0292
MRPL8	Mitochondrial Ribosomal Protein, Large subunit	-0.0464	0.0294
ISM1	Isoleucyl tRNA Synthetase of Mitochondria	-0.0322	0.0297
MAM33	Mitochondrial Acidic Matrix protein	-0.0566	0.0298
PET9	PETite	-0.0268	0.0303
HHT2	Histone H Three	-0.0291	0.0303
MRPL10	Mitochondrial Ribosomal Protein, Large subunit	-0.0422	0.0310
		-0.0281	0.0315
MRPL1	Mitochondrial Ribosomal Protein, Large subunit	-0.0311	0.0322
MRPL17	Mitochondrial Ribosomal Protein, Large subunit	-0.0279	0.0324
SCW10	Soluble Cell Wall protein	-0.0475	0.0334
FUM1	FUMarase	-0.0276	0.0335
WSC2	cell Wall integrity and Stress response Component	-0.0275	0.0338
YFH7		-0.0271	0.0341
SNZ2	SNooZe	-0.0604	0.0347
MRP1	Mitochondrial Ribosomal Protein	-0.0393	0.0351
QCR6	ubiQuinol-cytochrome C oxidoReductase	-0.0258	0.0355
KGD1	alpha-KetoGlutarate Dehydrogenase	-0.0251	0.0356
		-0.0266	0.0362
IFM1	Initiation Factor of Mitochondria	-0.0309	0.0365
ACP1	Acyl Carrier Protein	-0.0284	0.0368
MRPL3	Mitochondrial Ribosomal Protein, Large subunit	-0.0353	0.0374
MRPL13	Mitochondrial Ribosomal Protein, Large subunit	-0.0427	0.0382
NRM1	Negative Regulator of MBF targets	-0.0264	0.0386
MRPL15	Mitochondrial Ribosomal Protein, Large subunit	-0.0377	0.0388
MRPL37	Mitochondrial Ribosomal Protein, Large subunit	-0.0391	0.0392
PRY2	Pathogen Related in Yeast	-0.0485	0.0394
PCK1	Phosphoenolpyruvate CarboxyKinase	-0.0300	0.0396
CYC1	CYtochrome C	-0.0526	0.0402
SIM1	Start Independent of Mitosis	-0.0457	0.0402
AIM17	Altered Inheritance rate of Mitochondria	-0.0371	0.0406

TUB1	TUBulin	-0.0282	0.0409
SRL1	Suppressor of Rad53 null Lethality	-0.0645	0.0410
MRPL6	Mitochondrial Ribosomal Protein, Large subunit	-0.0316	0.0415
SSC1	Stress-Seventy subfamily C	-0.0268	0.0424
MRP51	Mitochondrial Ribosomal Protein	-0.0269	0.0438
IRC14		-0.0261	0.0439
SMC3	Stability of MiniChromosomes	-0.0273	0.0447
MRP49	Mitochondrial Ribosomal Protein	-0.0280	0.0449
MRPL11	Mitochondrial Ribosomal Protein, Large subunit	-0.0381	0.0450
TOM20	Translocase of the Outer Mitochondrial membrane	-0.0308	0.0450
YNK1	Yeast Nucleoside diphosphate Kinase	-0.0350	0.0450
MCD1	Mitotic Chromosome Determinant	-0.0293	0.0459
MRPL24	Mitochondrial Ribosomal Protein, Large subunit	-0.0395	0.0460
AIM24	Altered Inheritance rate of Mitochondria	-0.0284	0.0468
RSM18	Ribosomal Small subunit of Mitochondria	-0.0271	0.0469
CYM1	CYtosolic Metalloprotease	-0.0288	0.0473
GCG1	Gamma-glutamyl Cyclotransferase acting on Glutathione	-0.0259	0.0476
SUC2	SUCrose	-0.0391	0.0476
RSM7	Ribosomal Small subunit of Mitochondria	-0.0321	0.0478
MSW1	Mitochondrial aminoacyl-tRNA Synthetase, tryptophan (W)	-0.0293	0.0481
DPC29	Delta-Psi dependent mitochondrial import and Cleavage protein of ~29 kDa	-0.0386	0.0481
YOX1	Yeast homeobOX	-0.0346	0.0484
IBA57	Iron-sulfur cluster assembly factor for Biotin synthase and Aconitase-like mitochondrial proteins	-0.0311	0.0486
ACH1	Acetyl CoA Hydrolase	-0.0507	0.0492
FMC1	Formation of Mitochondrial Complexes	-0.0379	0.0495

Table S8. Significant TFs of eigen-phase 1A.

TF	Class	P-value
Tod6	Tryptophan cluster factors	0
Dot6	Tryptophan cluster factors	0

Sfp1	C2H2 zinc finger factors	0
Stb3	Unknown	0
Sum1	A.T hook factors	0
Lys14	C6 zinc cluster factors	0.0003
Rgt1	C6 zinc cluster factors	0.0005
YKL222C	C6 zinc cluster factors	0.0006
YNR063W	C6 zinc cluster factors	0.0027
Ert1	C6 zinc cluster factors	0.0047
Azf1	C2H2 zinc finger factors	0.0052
Hal9	C6 zinc cluster factors	0.0062
Yrr1	C6 zinc cluster factors	0.0068
Pdr8	C6 zinc cluster factors	0.0077
Met28	Basic leucine zipper factors (bZIP)	0.0117
Ecm22	C6 zinc cluster factors	0.0121
Urc2	C6 zinc cluster factors	0.0145
Aro80	C6 zinc cluster factors	0.0159
Put3	C6 zinc cluster factors	0.0169
Xbp1	Unknown	0.0174
Pdr1	C6 zinc cluster factors	0.0177
YPR196W	C6 zinc cluster factors	0.018
Cat8	C6 zinc cluster factors	0.0254
Bas1	Tryptophan cluster factors	0.029
Met4	Basic leucine zipper factors (bZIP)	0.0294
Rme1	C2H2 zinc finger factors	0.0318
Rdr1	C6 zinc cluster factors	0.032
Hap1	C6 zinc cluster factors	0.0339
Rap1	Tryptophan cluster factors	0.0363
Uga3	C6 zinc cluster factors	0.0375
Yrm1	C6 zinc cluster factors	0.0399
Ecm23	Other C4 zinc finger-type factors	0.0445
Cup2	Copper-fist DNA-binding domain	0.0455
Gsm1	C6 zinc cluster factors	0.0491

Table S9. Significant TFs of eigen-phase 1B.

TF	Class	P-value
Adr1	C2H2 zinc finger factors	0
Cat8	C6 zinc cluster factors	0
Crz1	C2H2 zinc finger factors	0
Gis1	C2H2 zinc finger factors	0
Mig2	C2H2 zinc finger factors	0

Mig3	C2H2 zinc finger factors	0
Msn2	C2H2 zinc finger factors	0
Msn4	C2H2 zinc finger factors	0
Nhp10	High-mobility group (HMG) domain factors	0
Rei1	C2H2 zinc finger factors	0
Rgm1	C2H2 zinc finger factors	0
Rph1	C2H2 zinc finger factors	0
Rsc30	C6 zinc cluster factors	0
Sut1	C6 zinc cluster factors	0
Usv1	C2H2 zinc finger factors	0
Urc2	C6 zinc cluster factors	0
YER130C	C2H2 zinc finger factors	0
YGR067C	C2H2 zinc finger factors	0
Tda9	C2H2 zinc finger factors	0
Zms1	C2H2 zinc finger factors	0
Mig1	C2H2 zinc finger factors	0.0001
Uga3	C6 zinc cluster factors	0.0001
YLL054C	C6 zinc cluster factors	0.0001
Azf1	C2H2 zinc finger factors	0.0002
Oaf1	C6 zinc cluster factors	0.0002
Hap1	C6 zinc cluster factors	0.0003
Cha4	C6 zinc cluster factors	0.0004
Sip4	C6 zinc cluster factors	0.0004
Ash1	Other C4 zinc finger-type factors	0.0006
YER184C	C6 zinc cluster factors	0.0006
YNR063W	C6 zinc cluster factors	0.0006
Dal81	C6 zinc cluster factors	0.0007
Tea1	C6 zinc cluster factors	0.0007
Rpn4	C2H2 zinc finger factors	0.0009
Stb5	C6 zinc cluster factors	0.0009
Stp2	C2H2 zinc finger factors	0.0012
Pdr1	C6 zinc cluster factors	0.0013
Rsc3	C6 zinc cluster factors	0.0014
Tbs1	C6 zinc cluster factors	0.0017
YLR278C	C6 zinc cluster factors	0.0018
Gal4	C6 zinc cluster factors	0.0027
Sut2	C6 zinc cluster factors	0.0032
Pdr3	C6 zinc cluster factors	0.0042
Yrr1	C6 zinc cluster factors	0.0043
Gsm1	C6 zinc cluster factors	0.0045
Rds1	C6 zinc cluster factors	0.0054
Stp1	C2H2 zinc finger factors	0.0069

Ime1	Unknown	0.0071
Rdr1	C6 zinc cluster factors	0.0095
Ecm22	C6 zinc cluster factors	0.0109
Ndt80	NDT80 domain factors	0.0126
Ume6	C6 zinc cluster factors	0.0131
Cep3	C6 zinc cluster factors	0.0136
Hal9	C6 zinc cluster factors	0.015
Dot6	Tryptophan cluster factors	0.0156
Snt2	Tryptophan cluster factors	0.0183
YPR022C	C2H2 zinc finger factors	0.0259
YKL222C	C6 zinc cluster factors	0.0264
YPR196W	C6 zinc cluster factors	0.0268
YPR015C	C2H2 zinc finger factors	0.0272
Rds2	C6 zinc cluster factors	0.0283
Ert1	C6 zinc cluster factors	0.0287
Cst6	Basic leucine zipper factors (bZIP)	0.03
Sko1	Basic leucine zipper factors (bZIP)	0.0302
Aft2	Unknown	0.031
Nrg1	C2H2 zinc finger factors	0.0333
Opi1	Unknown	0.0387
Srd1	Other C4 zinc finger-type factors	0.0406
Arg80	MADS box factors	0.044

Table S10. Significant TFs of eigen-phase 2A.

TF	Class	P-value
Tod6	Tryptophan cluster factors	0
Dot6	Tryptophan cluster factors	0
Sfp1	C2H2 zinc finger factors	0
Stb3	Unknown	0.0001
Swi5	A.T hook factors	0.0011
Gis1	C2H2 zinc finger factors	0.0018
Mcm1	MADS box factors	0.0049
Ash1	Other C4 zinc finger-type factors	0.0057
YNR063W	C6 zinc cluster factors	0.0059
Azf1	C2H2 zinc finger factors	0.0066
Rph1	C2H2 zinc finger factors	0.0072
Sfl1	Heat shock factors	0.0106
Rgm1	C2H2 zinc finger factors	0.0115
Msn2	C2H2 zinc finger factors	0.0119

Msn4	C2H2 zinc finger factors	0.012
YKL222C	C6 zinc cluster factors	0.0123
Sum1	A.T hook factors	0.0183
YER130C	C2H2 zinc finger factors	0.0211
Spt2	Unknown	0.0246
Tbs1	C6 zinc cluster factors	0.0251
Zms1	C2H2 zinc finger factors	0.0298
Ace2	C2H2 zinc finger factors	0.0322
Hap1	C6 zinc cluster factors	0.0329
Cep3	C6 zinc cluster factors	0.0355
Reb1	Tryptophan cluster factors	0.037
Eds1	C6 zinc cluster factors	0.0371
YGR067C	C2H2 zinc finger factors	0.0376
YPR196W	C6 zinc cluster factors	0.0387
Ecm22	C6 zinc cluster factors	0.0406
Sut2	C6 zinc cluster factors	0.0453
Spt15	TATA-binding proteins	0.0454

Table S11. Significant TFs of eigen-phase 2B.

TF	Class	P-value
Rsc30	C6 zinc cluster factors	0.0004
Hap1	C6 zinc cluster factors	0.0014
Nhp10	High-mobility group (HMG) domain factors	0.0015
Hap3	Heteromeric CCAAT-binding factors	0.0028
Mig2	C2H2 zinc finger factors	0.0032
Spt15	TATA-binding proteins	0.0074
Hap2	Heteromeric CCAAT-binding factors	0.0078
Mig1	C2H2 zinc finger factors	0.0126
Sfp1	C2H2 zinc finger factors	0.013
Mig3	C2H2 zinc finger factors	0.0195
Rim101	C2H2 zinc finger factors	0.0235
Arg80	MADS box factors	0.0246
Hap5	Heteromeric CCAAT-binding factors	0.0284
Sut2	C6 zinc cluster factors	0.0305
Asg1	C6 zinc cluster factors	0.0318
Sip4	C6 zinc cluster factors	0.0405
Abf2	High-mobility group (HMG) domain factors	0.041
Put3	C6 zinc cluster factors	0.0464
Zms1	C2H2 zinc finger factors	0.0466

References

1. Kuang, Z., Cai, L., Zhang, X., Ji, H., Tu, B.P. and Boeke, J.D. (2014) High-temporal-resolution view of transcription and chromatin states across distinct metabolic states in budding yeast. Nat Struct Mol Biol, 21, 854-863.
2. Tu, B.P., Kudlicki, A., Rowicka, M. and McKnight, S.L. (2005) Logic of the yeast metabolic cycle: temporal compartmentalization of cellular processes. Science, 310, 1152-1158.
3. Cao, S., Wang, L., Feng, Y., Peng, X.D. and Li, L.M. (2023) A data integration approach unveils a transcriptional signature of type 2 diabetes progression in rat and human islets. PLoS One, 18, e0292579.
4. Tu, B.P., Mohler, R.E., Liu, J.C., Dombek, K.M., Young, E.T., Synovec, R.E. and McKnight, S.L. (2007) Cyclic changes in metabolic state during the life of a yeast cell. Proc Natl Acad Sci U S A, 104, 16886-16891.
5. Marshall, R.S. and Vierstra, R.D. (2018) Autophagy: The Master of Bulk and Selective Recycling. Annu Rev Plant Biol, 69, 173-208.
6. Locatelli, A.G. and Cenci, S. (2022) Autophagy and longevity: Evolutionary hints from hyper-longevous mammals. Front Endocrinol (Lausanne), 13, 1085522.
7. Tanaka, K. (2009) The proteasome: overview of structure and functions. Proc Jpn Acad Ser B Phys Biol Sci, 85, 12-36.
8. Zhu, C., Byers, K.J., McCord, R.P., Shi, Z., Berger, M.F., Newburger, D.E., Saulrieta, K., Smith, Z., Shah, M.V., Radhakrishnan, M. et al. (2009) High-resolution DNA-binding specificity analysis of yeast transcription factors. Genome Res, 19, 556-566.
9. Liko, D., Slattery, M.G. and Heideman, W. (2007) Stb3 binds to ribosomal RNA processing element motifs that control transcriptional responses to growth in Saccharomyces cerevisiae. J Biol Chem, 282, 26623-26628.
10. Marion, R.M., Regev, A., Segal, E., Barash, Y., Koller, D., Friedman, N. and O'Shea, E.K. (2004) Sfp1 is a stress- and nutrient-sensitive regulator of ribosomal protein gene expression. Proc Natl Acad Sci U S A, 101, 14315-14322.
11. Knight, B., Kubik, S., Ghosh, B., Bruzzone, M.J., Geertz, M., Martin, V., Denervaud, N., Jacquet, P., Ozkan, B., Rougemont, J. et al. (2014) Two distinct promoter architectures centered on dynamic nucleosomes control ribosomal protein gene transcription. Genes Dev, 28, 1695-1709.
12. Mivelaz, M., Cao, A.M., Kubik, S., Zencir, S., Hovius, R., Boichenko, I.,

Stachowicz, A.M., Kurat, C.F., Shore, D. and Fierz, B. (2020) Chromatin Fiber Invasion and Nucleosome Displacement by the Rap1 Transcription Factor. Mol Cell, 77, 488-500 e489.

13. Lieb, J.D., Liu, X., Botstein, D. and Brown, P.O. (2001) Promoter-specific binding of Rap1 revealed by genome-wide maps of protein-DNA association. Nat Genet, 28, 327-334.

14. Thomas, D., Jacquemin, I. and Surdin-Kerjan, Y. (1992) MET4, a leucine zipper protein, and centromere-binding factor 1 are both required for transcriptional activation of sulfur metabolism in Saccharomyces cerevisiae. Molecular and Cellular Biology, 12, 1719-1727.

15. Feller, A., Dubois, E., Ramos, F. and Piérard, A. (1994) Repression of the genes for lysine biosynthesis in Saccharomyces cerevisiae is caused by limitation of Lys14-dependent transcriptional activation. Molecular and Cellular Biology, 14, 6411-6418.

16. Blaiseau, P.L. and Thomas, D. (1998) Multiple transcriptional activation complexes tether the yeast activator Met4 to DNA. EMBO J, 17, 6327-6336.

17. Daignan-Fornier, B. and Fink, G.R. (1992) Coregulation of purine and histidine biosynthesis by the transcriptional activators BAS1 and BAS2. Proc Natl Acad Sci U S A, 89, 6746-6750.

18. Kuras, L., Cherest, H., Surdin-Kerjan, Y. and Thomas, D. (1996) A heteromeric complex containing the centromere binding factor 1 and two basic leucine zipper factors, Met4 and Met28, mediates the transcription activation of yeast sulfur metabolism. The EMBO Journal, 15, 2519-2529.

19. Huang, H.L. and Brandriss, M.C. (2000) The regulator of the yeast proline utilization pathway is differentially phosphorylated in response to the quality of the nitrogen source. Mol Cell Biol, 20, 892-899.

20. Sylvain, M.A., Liang, X.B., Hellauer, K. and Turcotte, B. (2011) Yeast zinc cluster proteins Dal81 and Uga3 cooperate by targeting common coactivators for transcriptional activation of gamma-aminobutyrate responsive genes. Genetics, 188, 523-534.

21. Iraqui, I., Vissers, S., Andre, B. and Urrestarazu, A. (1999) Transcriptional induction by aromatic amino acids in Saccharomyces cerevisiae. Mol Cell Biol, 19, 3360-3371.

22. Martínez-Pastor, M.T., Marchler, G., Schüller, C., Marchler-Bauer, A., Ruis, H. and Estruch, F. (1996) The Saccharomyces cerevisiae zinc finger proteins Msn2p and Msn4p are required for transcriptional induction through the stress response element (STRE). The EMBO Journal, 15, 2227-2235.

23. Larochelle, M., Drouin, S., Robert, F. and Turcotte, B. (2006) Oxidative

stress-activated zinc cluster protein Stb5 has dual activator/repressor functions required for pentose phosphate pathway regulation and NADPH production. Mol Cell Biol, 26, 6690-6701.

24. Baker, L.A., Ueberheide, B.M., Dewell, S., Chait, B.T., Zheng, D. and Allis, C.D. (2013) The yeast Snt2 protein coordinates the transcriptional response to hydrogen peroxide-mediated oxidative stress. Mol Cell Biol, 33, 3735-3748.

25. Rep, M., Proft, M., Remize, F., Tamas, M., Serrano, R., Thevelein, J.M. and Hohmann, S. (2001) The Saccharomyces cerevisiae Sko1p transcription factor mediates HOG pathway-dependent osmotic regulation of a set of genes encoding enzymes implicated in protection from oxidative damage. Mol Microbiol, 40, 1067-1083.

26. Blaiseau, P.L., Lesuisse, E. and Camadro, J.M. (2001) Aft2p, a novel iron-regulated transcription activator that modulates, with Aft1p, intracellular iron use and resistance to oxidative stress in yeast. J Biol Chem, 276, 34221-34226.

27. Vyas, V.K., Berkey, C.D., Miyao, T. and Carlson, M. (2005) Repressors Nrg1 and Nrg2 regulate a set of stress-responsive genes in Saccharomyces cerevisiae. Eukaryot Cell, 4, 1882-1891.

28. Zhang, X., Cui, Z., Miyakawa, T. and Moye-Rowley, W.S. (2001) Cross-talk between transcriptional regulators of multidrug resistance in Saccharomyces cerevisiae. J Biol Chem, 276, 8812-8819.

29. Hellauer, K., Akache, B., MacPherson, S., Sirard, E. and Turcotte, B. (2002) Zinc cluster protein Rdr1p is a transcriptional repressor of the PDR5 gene encoding a multidrug transporter. J Biol Chem, 277, 17671-17676.

30. Akache, B. and Turcotte, B. (2002) New regulators of drug sensitivity in the family of yeast zinc cluster proteins. J Biol Chem, 277, 21254-21260.

31. Akache, B., MacPherson, S., Sylvain, M.A. and Turcotte, B. (2004) Complex interplay among regulators of drug resistance genes in Saccharomyces cerevisiae. J Biol Chem, 279, 27855-27860.

32. Karunanithi, S. and Cullen, P.J. (2012) The filamentous growth MAPK Pathway Responds to Glucose Starvation Through the Mig1/2 transcriptional repressors in Saccharomyces cerevisiae. Genetics, 192, 869-887.

33. Dubacq, C., Chevalier, A. and Mann, C. (2004) The protein kinase Snf1 is required for tolerance to the ribonucleotide reductase inhibitor hydroxyurea. Mol Cell Biol, 24, 2560-2572.

34. Hlynialuk, C., Schierholtz, R., Vernooy, A. and van der Merwe, G. (2008) Nsf1/Ypl230w participates in transcriptional activation during non-fermentative growth and in response to salt stress in Saccharomyces cerevisiae. Microbiology (Reading), 154, 2482-2491.

35. Bodvard, K., Jorhov, A., Blomberg, A., Molin, M. and Kall, M. (2013) The yeast transcription factor Crz1 is activated by light in a Ca2+/calcineurin-dependent and PKA-independent manner. PLoS One, 8, e53404.

36. Angus-Hill, M.L., Schlichter, A., Roberts, D., Erdjument-Bromage, H., Tempst, P. and Cairns, B.R. (2001) A Rsc3/Rsc30 zinc cluster dimer reveals novel roles for the chromatin remodeler RSC in gene expression and cell cycle control. Mol Cell, 7, 741-751.

37. Simon, M., Adam, G., Rapatz, W., Spevak, W. and Ruis, H. (1991) The Saccharomyces cerevisiae ADR1 gene is a positive regulator of transcription of genes encoding peroxisomal proteins. Molecular and Cellular Biology, 11, 699-704.

38. Turcotte, B., Liang, X.B., Robert, F. and Soontorngun, N. (2010) Transcriptional regulation of nonfermentable carbon utilization in budding yeast. FEMS Yeast Res, 10, 2-13.

39. Mannhaupt, G., Schnall, R., Karpov, V., Vetter, I. and Feldmann, H. (1999) Rpn4p acts as a transcription factor by binding to PACE, a nonamer box found upstream of 26S proteasomal and other genes in yeast. FEBS Lett, 450, 27-34.

40. Bartholomew, C.R., Suzuki, T., Du, Z., Backues, S.K., Jin, M., Lynch-Day, M.A., Umekawa, M., Kamath, A., Zhao, M., Xie, Z. et al. (2012) Ume6 transcription factor is part of a signaling cascade that regulates autophagy. Proc Natl Acad Sci U S A, 109, 11206-11210.

41. Orzechowski Westholm, J., Tronnersjo, S., Nordberg, N., Olsson, I., Komorowski, J. and Ronne, H. (2012) Gis1 and Rph1 regulate glycerol and acetate metabolism in glucose depleted yeast cells. PLoS One, 7, e31577.

42. Thepnok, P., Ratanakhanokchai, K. and Soontorngun, N. (2014) The novel zinc cluster regulator Tog1 plays important roles in oleate utilization and oxidative stress response in Saccharomyces cerevisiae. Biochem Biophys Res Commun, 450, 1276-1282.

43. Kassir, Y., Adir, N., Boger-Nadjar, E., Raviv, N.G., Rubin-Bejerano, I., Sagee, S. and Shenhar, G. (2003) Transcriptional regulation of meiosis in budding yeast. Int Rev Cytol, 224, 111-171.

44. Neiman, A.M. (2011) Sporulation in the budding yeast Saccharomyces cerevisiae. Genetics, 189, 737-765.

45. Morrow, B.E., Johnson, S.P. and Warner, J.R. (1989) Proteins That Bind to the Yeast rDNA Enhancer. Journal of Biological Chemistry, 264, 9061-9068.

46. Bosio, M.C., Fermi, B., Spagnoli, G., Levati, E., Rubbi, L., Ferrari, R., Pellegrini, M. and Dieci, G. (2017) Abf1 and other general regulatory factors control ribosome biogenesis gene expression in budding yeast. Nucleic Acids

Res, 45, 4493-4506.

47. Messenguy, F. and Dubois, E. (1993) Genetic evidence for a role for MCM1 in the regulation of arginine metabolism in Saccharomyces cerevisiae. Mol Cell Biol, 13, 2586-2592.

48. Kuo, M.H. and Grayhack, E. (1994) A library of yeast genomic MCM1 binding sites contains genes involved in cell cycle control, cell wall and membrane structure, and metabolism. Mol Cell Biol, 14, 348-359.

49. Zitomer, R.S. and Lowry, C.V. (1992) Regulation of gene expression by oxygen in Saccharomyces cerevisiae. Microbiol Rev, 56, 1-11.

50. Cho, J.H., Lee, Y.K. and Chae, C.B. (2001) The modulation of the biological activities of mitochondrial histone Abf2p by yeast PKA and its possible role in the regulation of mitochondrial DNA content during glucose repression. Biochim Biophys Acta, 1522, 175-186.

51. Srinivasan, S., Torres, A.G. and Ribas de Pouplana, L. (2021) Inosine in Biology and Disease. Genes (Basel), 12.

52. Holecek, M. (2023) Roles of malate and aspartate in gluconeogenesis in various physiological and pathological states. Metabolism, 145, 155614.

53. Eisenberg, T., Knauer, H., Schauer, A., Buttner, S., Ruckenstuhl, C., Carmona-Gutierrez, D., Ring, J., Schroeder, S., Magnes, C., Antonacci, L. et al. (2009) Induction of autophagy by spermidine promotes longevity. Nat Cell Biol, 11, 1305-1314.

54. Thurston, J.H., Sherman, W.R., Hauhart, R.E. and Kloepper, R.F. (1989) myo-inositol: a newly identified nonnitrogenous osmoregulatory molecule in mammalian brain. Pediatr Res, 26, 482-485.

55. Visnjic, D., Lalic, H., Dembitz, V., Tomic, B. and Smoljo, T. (2021) AICAr, a Widely Used AMPK Activator with Important AMPK-Independent Effects: A Systematic Review. Cells, 10.

56. Sanchez-Gaya, V., Casani-Galdon, S., Ugidos, M., Kuang, Z., Mellor, J., Conesa, A. and Tarazona, S. (2018) Elucidating the Role of Chromatin State and Transcription Factors on the Regulation of the Yeast Metabolic Cycle: A Multi-Omic Integrative Approach. Front Genet, 9, 578.
